# A Loss-of-Function Screen for Phosphatases that Regulate Neurite Outgrowth Identifies PTPN12 as a Negative Regulator of TrkB Tyrosine Phosphorylation

**DOI:** 10.1371/journal.pone.0065371

**Published:** 2013-06-13

**Authors:** Malene Ambjørn, Véronique Dubreuil, Federico Miozzo, Fabienne Nigon, Bente Møller, Shohreh Issazadeh-Navikas, Jacob Berg, Michael Lees, Jan Sap

**Affiliations:** 1 Department of Biomedical Sciences and Biotechnology Research and Innovation Centre (BRIC), Faculty of Health Sciences, University of Copenhagen, Copenhagen, Denmark; 2 Epigenetics and Cell Fate, University of Paris, Sorbonne Paris Cité, Paris, France; 3 Department of Wind Energy, Technical University of Denmark, Roskilde, Denmark; The Nathan Kline Institute, United States of America

## Abstract

Alterations in function of the neurotrophin BDNF are associated with neurodegeneration, cognitive decline, and psychiatric disorders. BDNF promotes axonal outgrowth and branching, regulates dendritic tree morphology and is important for axonal regeneration after injury, responses that largely result from activation of its tyrosine kinase receptor TrkB. Although intracellular neurotrophin (NT) signaling presumably reflects the combined action of kinases and phosphatases, little is known about the contributions of the latter to TrkB regulation. The issue is complicated by the fact that phosphatases belong to multiple independently evolved families, which are rarely studied together. We undertook a loss-of-function RNA-interference-based screen of virtually all known (254) human phosphatases to understand their function in BDNF/TrkB-mediated neurite outgrowth in differentiated SH-SY5Y cells. This approach identified phosphatases from diverse families, which either positively or negatively modulate BDNF-TrkB-mediated neurite outgrowth, and most of which have little or no previously established function related to NT signaling. “Classical” protein tyrosine phosphatases (PTPs) accounted for 13% of the candidate regulatory phosphatases. The top classical PTP identified as a negative regulator of BDNF-TrkB-mediated neurite outgrowth was PTPN12 (also called PTP-PEST). Validation and follow-up studies showed that endogenous PTPN12 antagonizes tyrosine phosphorylation of TrkB itself, and the downstream activation of ERK1/2. We also found PTPN12 to negatively regulate phosphorylation of p130cas and FAK, proteins with previously described functions related to cell motility and growth cone behavior. Our data provide the first comprehensive survey of phosphatase function in NT signaling and neurite outgrowth. They reveal the complexity of phosphatase control, with several evolutionarily unrelated phosphatase families cooperating to affect this biological response, and hence the relevance of considering all phosphatase families when mining for potentially druggable targets.

## Introduction

During development, neurons extend neurites in response to limited amounts of soluble or insoluble signals [1]. One of the neurites will eventually become the axon and extend for a long way to reach its target, while the remaining neurites will become dendrites [2]. Axon elongation depends on the structure and dynamics of actin filaments and microtubules within the growth cone, which is strictly regulated by intracellular signaling cascades in response to extracellular stimuli including growth factors, such as neurotrophins (NTs) and extracellular matrix [1], [3], [4].

NTs are a major group of neurotrophic factors, which regulate numerous neuronal functions during development, in the adult state and in response to injury, such as neuronal survival and death, cell migration, axon growth, synaptogenesis, neuronal transmission and synaptic plasticity [1], [5]–[7]. Members of the NT family include nerve-growth factor (NGF), brain-derived neurotrophic factor (BDNF), NT-3 and NT-4/5. NTs bind to and signal through two types of receptors: tropomyosin-related kinase (Trk) receptors (NGF/TrkA), (BDNF and NT4-5/TrkB) and (NT-3/TrkC) [1]; and the non-kinase p75 neurotrophin receptor (p75^NTR^). Among the NTs, BDNF is enriched in the central nervous system (CNS) [6] as is its receptor, TrkB. BDNF promotes axon elongation and branching *in vitro* [8]–[10] and *in vivo* [9], and in addition regulates dendrite tree morphology including primary dendrite formation, dendrite branching and numbers of dendritic spines [9], [11]. Trk receptors are prototypical receptor-tyrosine kinases, which upon ligand-induced dimerization autophosphorylate tyrosine residues in their kinase domains as part of the activation process. Phosphorylated tyrosine residues of activated Trk receptors also function as docking sites for enzymes and scaffold proteins leading to activation of the downstream signaling pathways Ras-Raf-MEK-MAPK, PI3K-Akt and PLC-γ1-PKC [1], [12]. In relation to BDNF-TrkB signaling the first two have been associated with neuronal differentiation, neurite outgrowth and neuronal survival [1], [13], while the PLC-γ1-PKC pathway rather is involved in synaptic plasticity [14].

Importantly, TrkB activation can also occur in response to non-NT ligands such as glucocorticoids and adenosine as a consequence of engagement of heterologous receptors, a process known as transactivation [15], [16]. Furthermore, engagement of p75^NTR^ by NTs can either enhance or suppress Trk signalling, depending on the cellular context [17] and the degree of processing of the pro-form; engagement of a supramolecular receptor complex composed of TrkB, p75NTR and sortilin is reported to mediate apoptosis [5], [18], [19].

NTs also play important roles in the diseased and recovering nervous system. A common polymorphism in the human BNDF gene is genetically associated with risk of various psychiatric disorders [20]. As BDNF is a crucial modulator of synaptic plasticity, its proper function is of relevance for cognitive functions [21], [22]. Moreover, neurodegenerative conditions such as Alzheimer’s disease, Parkinson’s disease and Huntington’s disease are correlated with decreased levels of BDNF and are associated with a loss of synaptic connectivity and axonal demise [23], [24]. The BDNF-TrkB system also has a crucial role during nerve regeneration, as demonstrated in experiments where neutralizing antibodies to BDNF applied to animals with nerve lesion impede axonal outgrowth [25], or where mice heterozygous for the TrkB receptor show diminished axonal regeneration compared to wild-type animals following nerve transection [26]. In spite of their theoretical potential, NTs as therapeutic agents have had limited success due to poor pharmacokinetics [27] and severe side-effects [28], and thus treatment strategies improving cell intrinsic mechanisms of axon elongation and regeneration would be beneficial. Another recent strategy is the development of BDNF mimetics specifically enhancing TrkB signaling, which have showed promising results in rodent neurodegenerative models [29].

Intracellular signaling cascades depend on dynamic phosphorylation events that are tightly controlled by both kinases and phosphatases. The ability of non-specific protein tyrosine phosphatases (PTP) inhibitors to activate Trk signaling and promote neuronal outgrowth and survival *in vitro* [30], [31] and neuroprotection *in vivo* [32] points to a role for PTPs in regulation of Trk signaling. In accordance, RPTPζ (PTPRZ1) and RPTPσ (PTPRS) can dephosphorylate TrkA and suppress NGF-induced neurite outgrowth in PC12 cells and sensory neurons, respectively [33], [34]. Conversely, the RPTP LAR (PTPRF) augments TrkB-mediated signaling in hippocampal neurons through a mechanism involving dephosphorylation of Src and activation of TrkB [35]. Recently, the dual-specific phosphatase MKP-1 (DUSP1) was found to be required for BDNF-dependent axonal branching [36], emphasizing that phosphatases can have diverse functions in relation to Trk signaling. While the role of a few phosphatases in relation to neurite outgrowth and Trk signaling has been studied in detail, the majority has not been investigated.

Advances in large scale RNA interference (RNAi) screen techniques and complex phenotypic analysis have made it possible to take a more comprehensive approach in understanding the signaling events underlying specific cellular processes. In relation to neurite outgrowth this was recently exploited in a genome-wide screen using *Drosophila* primary neurons [37], and in two kinase screens related to retinoic acid (RA)-induced neurite outgrowth in SH-SY5Y neuroblastoma cells [38], [39].

In the present study we screened a highly comprehensive set (254 genes) of human genomic phosphatases for their potential to regulate BDNF/TrkB-mediated neurite outgrowth in an *in vitro* cell-based assay. We identified multiple phosphatases that either negatively or positively modulate neurite outgrowth. Mechanistic analysis of the negative modulation of neurite outgrowth by the protein tyrosine phosphatase PTPN12 (also known as PTP-PEST) showed that it acts as a negative regulator of tyrosine phosphorylation not only of p130cas and FAK as previously reported in other cells, but also of TrkB. Moreover, PTPN12 knockdown enhanced ERK1/2 activity (which is important during TrkB-mediated neurite outgrowth [1], [13]) in a TrkB-dependent manner.

## Results

### Sequential Treatment with Retinoic Acid and BDNF in SH-SY5Y Cells

**Figure 1:**
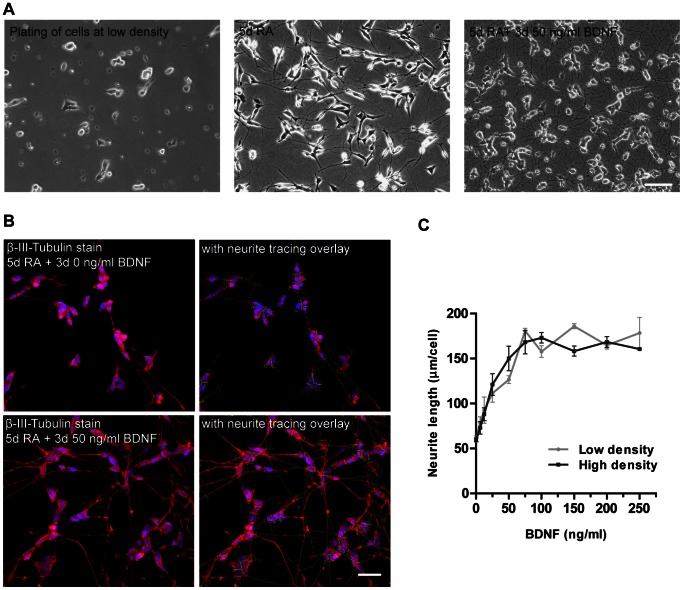
Automatic quantification of neurite outgrowth in RA/BDNF differentiated SH-SY5Y cells. A) Phase-contrast pictures of SH-SY5Y cells plated at low density (left picture) and cultured for 5 days with 10 µM RA (middle picture) followed by 3 days in 50 ng/ml (∼2 nM) BDNF in serum-free medium (right picture). Scale bar = 50 µm. B) Representative fluorescent pictures of SH-SY5Y cells stained with an anti-β-III-Tubulin antibody and Hoechst 33342. Cells were treated as in A) but with either 0 ng/ml BDNF (upper panel) or 50 ng/ml BDNF (lower panel). The pictures were acquired automatically using an IN Cell 1000 automated high throughput microscope with a Nikon 20× objective. Cells are shown without (left panel) or with (right panel) overlay of the neurite tracing program. Scale bar = 50 µm. C) BDNF-neurite length dose response curve at low or high cell densities (13,000 or 16,000 cells/cm^2^, respectively). Cells were differentiated for 5 days in RA and 3 days without or with BDNF at various concentrations (0–250 ng/ml), stained as in B), and evaluated for neurite outgrowth using the developed neurite outgrowth algorithm. Data are shown as mean ± S.E.M. of triplicates, and are representative of three individual experiments.

To identify phosphatases that regulate BDNF-TrkB-mediated neurite outgrowth we developed a functional siRNA-based screen system using the human neuroblastoma cell line SH-SY5Y [40]. After sequential treatment with retinoic acid (RA) and brain-derived neurotrophic factor BDNF SH-SY5Y cells faithfully mimic properties of differentiated neuron-like cells, as indicated by cell cycle withdrawal, dependence on BDNF, and expression of multiple neuronal and neuronal polarity markers [41], [42]. Treatment of naive SH-SY5Y cells for 4–5 days with RA resulted in a partly differentiated neuronal phenotype manifested by long neuritic processes and growth inhibition (Figure 1A) [41], [43]. RA induces the expression of TrkB with maximum expression after 5 days, thus sensitizing the cells to BDNF treatment [41]. Application of BDNF in serum-free medium to cells pretreated with RA for 5 days resulted in a burst of neurite outgrowth (Figure 1A) and increased level of GAP43 (Figure S1). GAP43 is a highly expressed protein within the neuronal growth cone that is important for growth and guidance of neurites [2], [44], and its level correlates positively with differentiation and the amount of neurite outgrowth [45], [46]. Under these conditions in the presence of BDNF cells can be maintained for more than 3 weeks without signs of neurodegeneration [41], thus giving a unique possibility to study BDNF-TrkB-mediated neuronal events such as neuronal survival and neurite outgrowth in a defined medium environment.

### Large-scale Loss of Function Screen of Human Phosphatases Involved in Neurite Outgrowth

To be able to estimate neurite outgrowth in an unbiased automatic manner in a high-throughput context we developed an algorithm which reliably could measure neurite length of β-III-Tubulin stained neurites across different neuronal densities and BDNF concentrations (Figures 1B and C). Cells pre-treated with RA responded to BDNF with neurite outgrowth in a dose-dependent manner, reaching saturation at 50 ng/ml BDNF (Figure 1C). We observed that this treatment induced neurites of lengths largely ranging between 50–200 µm, which is in accordance with what has been previously been published using these cells [38].

**Figure 2:**
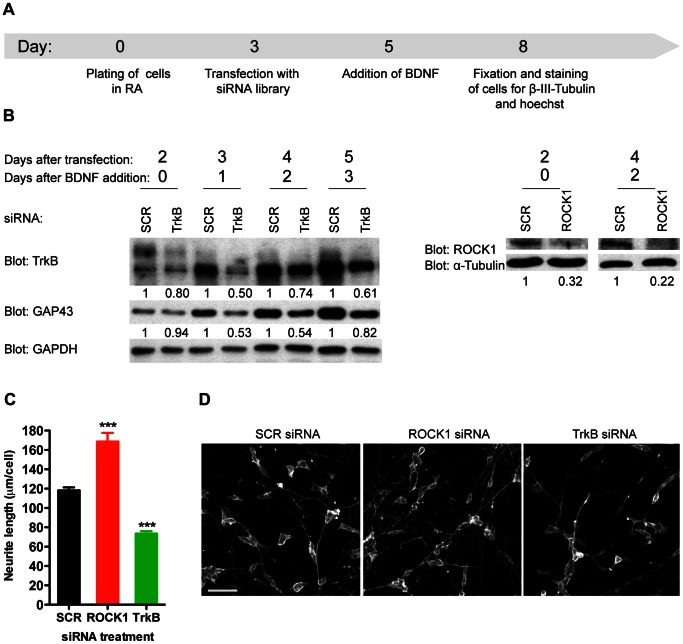
Set-up of siRNA-mediated loss-of-function phosphatase screen in differentiated SH-SY5Y cells. A) Assay outline for the phosphatase screen. Cells were plated in RA for a total of 5 days. At day 3 cells were transfected for 8 h with 50 nM siRNA in the presence of RA. At day five, 48 h post-transfection, cells were changed into serum-free medium with 10 ng/ml BDNF and left to differentiate for 3 more days. Then cells were fixed and stained with an anti-β-III-Tubulin antibody and Hoechst 33342. Image acquisition and data analysis were carried out automatically (see Materials and Methods). B) Using the set-up described, cells were transfected with scrambled (SCR) siRNA or siRNA targeting TrkB or ROCK1, treated with 50 ng/ml BDNF and lysed on different days as indicated. Expression levels of TrkB, GAP43 and ROCK1 were evaluated using western blotting. GAPDH or α-Tubulin was used as loading controls. Densitometric quantification of the relative protein expression levels is shown below the blots. C) Cells transfected and treated and evaluated as described in A). SCR, TrkB or ROCK1 siRNAs were used. Data are shown as mean and S.E.M. of four replicates and is representative of two independent experiments (***: p≤0.001; using one-way ANOVA followed by Dunnett’s multiple comparison test with SCR siRNA treated cells as a reference). D) Representative pictures of cells transfected with SCR, ROCK1 or TrkB specific siRNAs (day 8 of A). Scale bar = 50 µm.

To integrate the RA-BDNF system with high-throughput RNAi protocols we established transfection conditions using siRNAs targeting TrkB and Rho-associated, coiled-coil containing protein kinase 1 (ROCK1), which are well-established positive or negative modulators of neurite outgrowth, respectively [35], [41], [47]. With the aim being to screen for phosphatases implicated in BDNF-TrkB related signaling we designed an experimental setup wherein siRNA transfection after 3 days of RA would result in knockdown of target genes before BDNF addition (which occurs at day 5 of RA treatment), but with minimal interference of the RA-differentiation *per se*. Cells were then left to differentiate in a non-saturating concentration of BDNF (10 ng/ml, ∼0.35 nM) in serum-free medium for 3 more days, which would allow for identification of both positive and negative regulators of BDNF-TrkB dependent neurite outgrowth (Figure 2A).

Under these conditions the transfection efficiency was more than 97% (Figure S2). Efficient knockdown at the protein level of both TrkB and ROCK1 was seen at the time of BDNF addition and persisted throughout the BDNF treatment period (Figure 2B). Silencing of TrkB resulted in clear inhibition of GAP43 induction by BDNF (compared to cells transfected with a scrambled (SCR) siRNA control) (Figure 2B, left blot), indicating that it was possible to silence BDNF-TrkB signaling and to modulate downstream biological responses using our protocol. Importantly, transfection with siRNA targeting either ROCK1 or TrkB significantly increased or decreased BDNF-induced neurite outgrowth, respectively, compared to transfection with the SCR siRNA control (Figures 2C and D). No sequence-independent off-target effects (OTEs), potentially caused by introducing siRNAs into the cells [48]–[50], were seen when testing 6 different SCR control siRNAs in comparison to mock transfected or non-treated control cells at 3 different BDNF concentrations (Figure S3).

**Figure 3:**
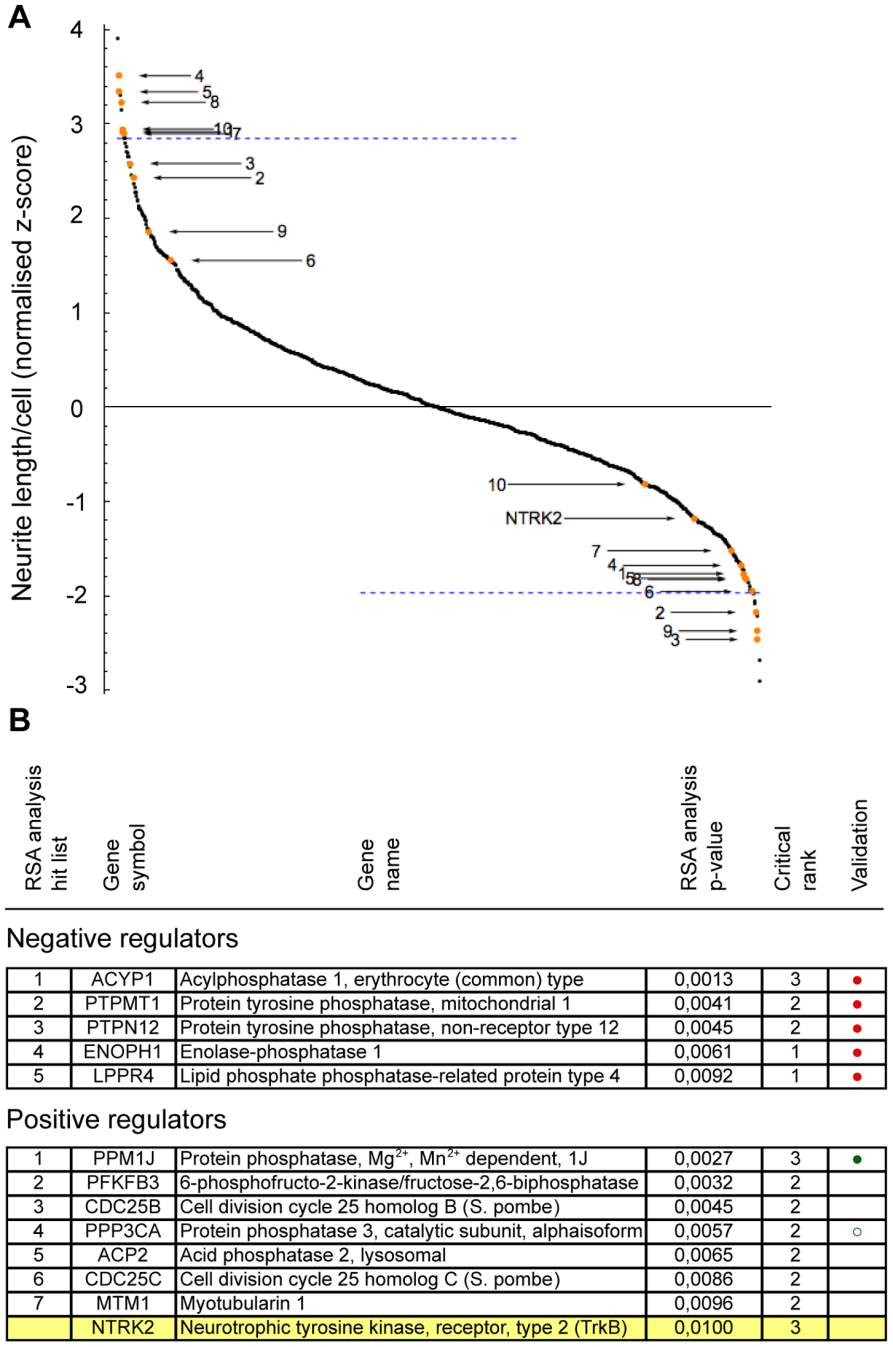
Primary screen data. A) Neurite length after siRNA-mediated knockdown of 254 different human phosphatases. Activities of individual siRNAs are plotted as a function of the standard deviation of the median screen activity (*z*-score) (with each value representing the average from three independent screens). The blue dashed lines mark the “top 10″ activity cut-off for either negative regulators (above zero) or positive regulators (below zero) of neurite outgrowth, while orange dots represent “top 10″ RSA hits (only the most potent siRNA for the particular gene is marked). B) RSA hit list including genes with p≤0.01 in the RSA analysis for either negative or positive regulators of neurite outgrowth. Critical rank indicates how many of the three gene-specific siRNAs contributed to the gene p-value. Filled circles represent hits that have been validated in a separate sub-screen, while open circles represent hits that could not be validated.

For the screens we designed a comprehensive library targeting 254 human phosphatases (including protein, lipid, and carbohydrate phosphatases). For each target gene, 3 different siRNAs (Ambion) were transfected (separately). The full screen was performed 3 independent times using different passages of the SH-SY5Y cells. The normalized activity values (neurite length/cell) obtained for individual sequence-specific siRNAs were averaged over the screens, generating one activity-value per siRNA, which was used for subsequent statistical analysis. This approach was undertaken to reduce random error, thus increasing the sensitivity of the screen data [51]. Data were normally distributed and calculated *z*-scores (standard deviation from median screen activity) for all siRNAs are plotted in Figure 3A.

### Analysis of Screen Data

The widespread use of high-throughput siRNA-based screens has highlighted the unfortunate caveat of a high incidence of sequence-dependent OTEs, resulting in false positive hits [52]–[54]. Conventional analysis methods are based on activity ranking followed by an arbitrary threshold cut-off selection process, which typically identifies hits with only very high activity. By contrast, redundant siRNA (RSA) analysis [55] is an alternative probability-based analysis approach, that takes into account the collective activity of all siRNAs targeting a specific gene, thus strongly reducing the chance of sequence-dependent OTEs [49], [55]. The method assigns a p-value to individual genes, which reflect the distribution of all the gene-specific siRNAs tested toward high (or low) activity. This type of analysis thus identifies hits that are distributed much deeper into the dataset than conventional activity cut-off methods while simultaneously yielding significantly better validation rates [55], which reflects a higher rate of identification of true biological hits in the primary analysis. In our dataset top 10 hits identified by RSA analysis also distributed much deeper into the dataset (orange dots) compared to top 10 activity hits (blue line, Figure 3A).

Figure 3B lists hits with a p-value ≤0.01 in the RSA analysis for either negative or positive regulators (extended hit lists for negative and positive regulators can be found in Tables S1 and S2, respectively). The NTRK2/TrkB and ROCK1 controls yielded p-values of 1.4E-12 and 2.0E-06, respectively, indicating that the screen performed well in both directions. These controls are not included in the hit list of identified regulators, since their p-values are not directly comparable to the tested genes due to their high number and pre-validated function (see Supporting Materials and Methods Document S1). However, three siRNAs targeting NTRK2, were also included as perfectly comparable controls in the pre-spotted siRNA plates from the manufacturer (Ambion), and thus can be compared directly to the other siRNAs in the library. In this case, we found that the RSA analysis placed NTRK2/TrkB among the top 10 positive regulators of BDNF-mediated outgrowth (p-value = 0.01), further supporting that the screen behaved satisfactorily.

### Validation

11 negative and 4 positive hits were selected for validation based on criteria of interest and representation of the various phosphatase subgroups. We predominantly selected negative modulators for validation and follow-up because their knockdown phenotype is more likely to be biologically specific, and in view of a potential interest as therapeutic targets.

**Figure 4:**
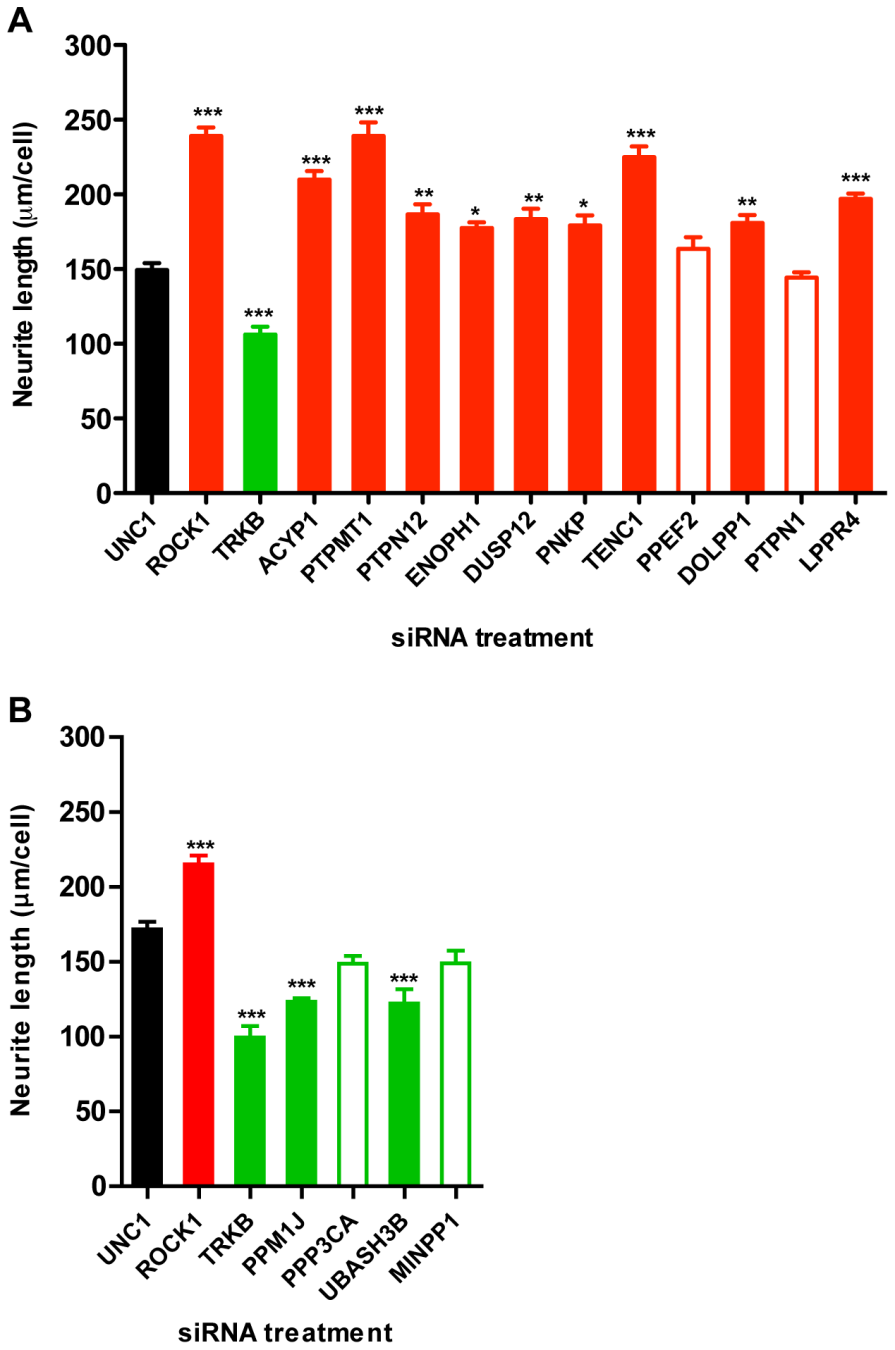
Validation of hits from the primary screen. Some of the hits identified in the main screen (here defined as p<0.05) were tested for validation in a sub-screen following the screen protocol described in Figure 2A using newly purchased siRNAs from another vendor. UNC1 is the scrambled siRNA control while ROCK1 or TrkB targeting siRNAs served as biological controls. A) 11 negative regulators and B) 4 positive regulators were included in the sub-screen. Negative and positive regulators were screened on separate plates, which both included controls. Normalization between plates was not performed, explaining the slight variation in the control values between A) and B). Red and green indicates negative and positive regulators respectively, and filled and open bars represent validated and non-validated hits, respectively. Data are shown as mean and S.E.M. of triplicates (*: p≤0.05; **: p≤0.01; and ***: p≤0.001; using one-way ANOVA followed by Dunnett’s multiple comparison test with UNC1 as reference).

The validation screen was performed essentially as for the main screens (10 ng/ml BDNF), using siRNAs corresponding in sequence to one of the three siRNAs in the main screen, but purchased from another vendor (Sigma). 9/11 of the negative regulators and 2/4 of the positive regulators tested were validated resulting in an overall validation efficiency of 73% (Figure 4). The knockdown efficiency of four randomly chosen hits, including both validated and non-validated hits was tested by qPCR (Figure S4). For all four genes specific siRNA treatment resulted in 50–75% reduction in mRNA level, which indicates a reasonable knockdown efficiency, and thus that lack of phenotypic validation did not necessarily correlate with lack of mRNA knockdown (PTPN1; Figure 4A).

Even though we purposely designed the screen to enrich for hits affecting BDNF signaling we considered the possibility that some hits would function in a BDNF- or TrkB-independent fashion. To distinguish between BDNF-dependent and -independent hits we performed further validation in absence of BDNF. Surprisingly, we observed a significant effect of TrkB knockdown on neurite outgrowth in the absence of BDNF (Figure S5A and B). This may reflect an autocrine source of BDNF [56], spontaneous dimerization of TrkB [57] or TrkB transactivation by other signals [58], [59], thus hampering assessment of hits in the absence of TrkB signaling. At the same time, this observation underscores the central contribution of TrkB signaling to outgrowth in our screening assay. Among the negative regulators, knockdown of some (ACYP1, PTPMT1 and TENC1) induced outgrowth even in absence of BDNF (Figure S5A), suggesting that they are either particularly important in the suppression of basal levels of TrkB signaling, or act on a parallel pathway; by contrast, knockdown of PTPN12 (and ENOPH1, DUSP12, PNKP, DOLPP1, LPPR4) did not affect outgrowth in SH-SY5Y cells in the absence of BDNF. Neither of the two validated positive regulators, PPM1J and UBASH3B, significantly affected outgrowth in absence of BDNF (Figure S5B), suggesting that either the BDNF-independent window for decreasing outgrowth was too small, or that outgrowth in absence of exogenous BDNF does not depend on these genes.

As opposed to the intermediate BDNF concentration used in the primary screen we also tested how knockdown of the identified hits would affect outgrowth in the presence of a saturating BDNF concentration (50 ng/ml) (Figure S5C and D). Among the negative regulators only PTPMT1 knockdown resulted in increased outgrowth even at saturating BDNF concentration, while others only suppressed outgrowth under modest BDNF concentrations (PTPN12, DUSP12, LPPR4) (Figure S5C). Under this condition loss of PPM1J but not UBASH3 significantly decreased neurite length, indicating that loss of PPM1J function cannot be rescued by higher BDNF concentration; by contrast UBASH3B appeared to be most critical for outgrowth at intermediate levels of BDNF (Figure S5D). These differential interactions with BDNF stimulation reveal a functional classification of protein phosphatases affecting neurite outgrowth.

### Hits Identified in the Screen

Among the genes identified are members of all phosphatase subgroups. ACYP1 is an acylphosphatase; ENOPH1 and PNKP belong to the l–2-halo-acid dehalogenase (HAD) superfamily of hydrolases; DOLPP1, LPPR4 and TENC1 are generally considered as lipid phosphatases, while PTPN12 is a classical tyrosine phosphatase. DUSP12 is a member of the dual-specific phosphatase family, while PPM1J is a serine/threonine phosphatase of the PPM family. Most of the genes identified have not previously been associated with a neurite outgrowth phenotype and many have been poorly characterized in the literature.

### PTPN12 Phenotypic Validation Using TrkB-SH-SY5Y Cells

PTPN12, which we identified as a negative regulator of BDNF-TrkB mediated neurite outgrowth (Figure 4A), is known to control various aspects of cell motility in response to both extracellular matrix (ECM) and growth factors in fibroblasts [60]–[63]. Recently, PTPN12 was found to interact with and dephosphorylate the RTKs EGFR and ErbB-2, thereby acting as a tumor suppressor in breast cancer by suppressing MAPK signaling [64]. Moreover, PTPN12 was found to interact with multiple RTKs such as PDGFβ, EGFR and c-Kit in HEK293 cells [65]. Furthermore, PTPN12 negatively regulates insulin-induced MAPK signaling through a direct interaction with the adaptor protein Shc, which leads to dephosphorylation of ShcY317 [66]. A similar mechanism for PTPN12 is reported in a B-cell line after BCR activation [67]. Strikingly, only a single study (on its negative regulatory role in response to Aβ-mediated neuronal dystrophy) has addressed the role of PTPN12 in a neuronal context [68]. This knowledge gap led us to examine PTPN12 function of in BDNF/TrkB-mediated neurite outgrowth in greater detail.

**Figure 5:**
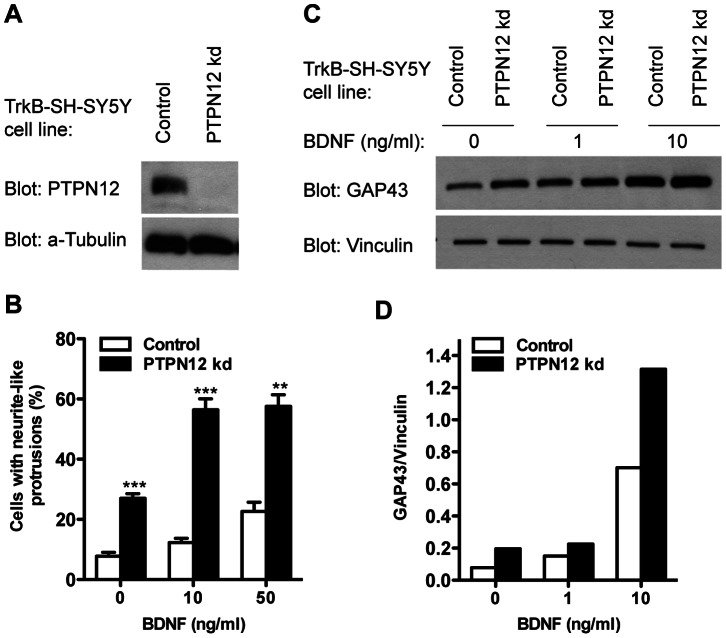
PTPN12 decreases neurite-like outgrowth in TrkB-SH-SY5Y cells. A) Western blot analysis of PTPN12 expression level in TrkB-SH-SY5Y cells stably expressing a pGIPZ shRNAmir non-targeting PTPN12 (PTPN12 kd) or the non-silencing control. α-Tubulin is used as reference. B) Neurite-like outgrowth in PTPN12 kd cells compared to the control. Cells were seeded at low density and grown for 24 h before stimulation with BDNF as indicated for 3 days in serum-free medium. Cells were then stained with a β-III-Tubulin antibody and Hoechst 33342 and pictures were acquired and analyzed automatically. Data shown are mean and S.E.M. of triplicates and are representative of two independent experiments. Statistical analysis was performed using Student’s t-test comparing PTPN12 kd and control cells for each BDNF concentration (**: p≤0.01; ***: p≤0.001). C) Western blot analysis of cells treated as in B) for GAP43 expression using Vinculin as loading reference. D) Densitometric quantification of the results in C).

For these studies we generated stable cell lines expressing a shRNAmir targeting PTPN12, or a non-silencing control, in SH-SY5Y cells that already stably express TrkB (TrkB-SH-SY5Y) [69] (Figure 5A). This TrkB-expressing cellular context was used to circumvent any effects of PTPN12 knockdown on differentiation with RA, thus securing that we only examined its effects on TrkB signaling. The TrkB-SH-SY5Y cells expressed levels of TrkB approximately similar to whole brain tissue and cultured cerebellar granule neurons (Figure S6A), which indicates a physiological relevance. The cells also responded to BDNF with neurite-like outgrowth and increased GAP43 expression (Figures S6B and C, respectively). However, BDNF-mediated outgrowth in TrkB-SH-SY5Y cells appeared morphologically distinct from that in RA-pretreated cells (compare Figure 1A with Figure S6B) indicating a contribution of RA-induced differentiation prior to BDNF stimulation. Nevertheless, we found that also in TrkB-SH-SY5Y cells not exposed to RA, PTPN12 knockdown increased the number of cells with neurite-like protrusions (a comparable but more sensitive parameter in TrkB-SH-SY5Y cells than mean neurite length/cell) in response to BDNF (Figure 5B). These morphological data were further strengthened by increased levels of GAP43 in the PTPN12 knockdown cells compared to controls (Figures 5C and D). We note that, as opposed to SH-SY5Y cells (Figure S5B), PTPN12 knockdown affected outgrowth in TrkB-SH-SY5Y cells even in absence of BDNF. However this does not exclude regulation of TrkB signaling by BDNF, particularly since the higher TrkB levels in TrkB-SH-SY5Y cells (Figure S6A) may favor ligand-independent TrkB signaling [34], [57]. We conclude that PTPN12 knockdown affects neurite outgrowth independently of a RA-induced differentiation response, and that TrkB-SH-SY5Y cells can be used to identify PTPN12 substrates that may be important for outgrowth, including potentially TrkB itself.

### PTPN12 Knockdown Causes Increased Phosphorylation of p130cas and FAK

**Figure 6:**
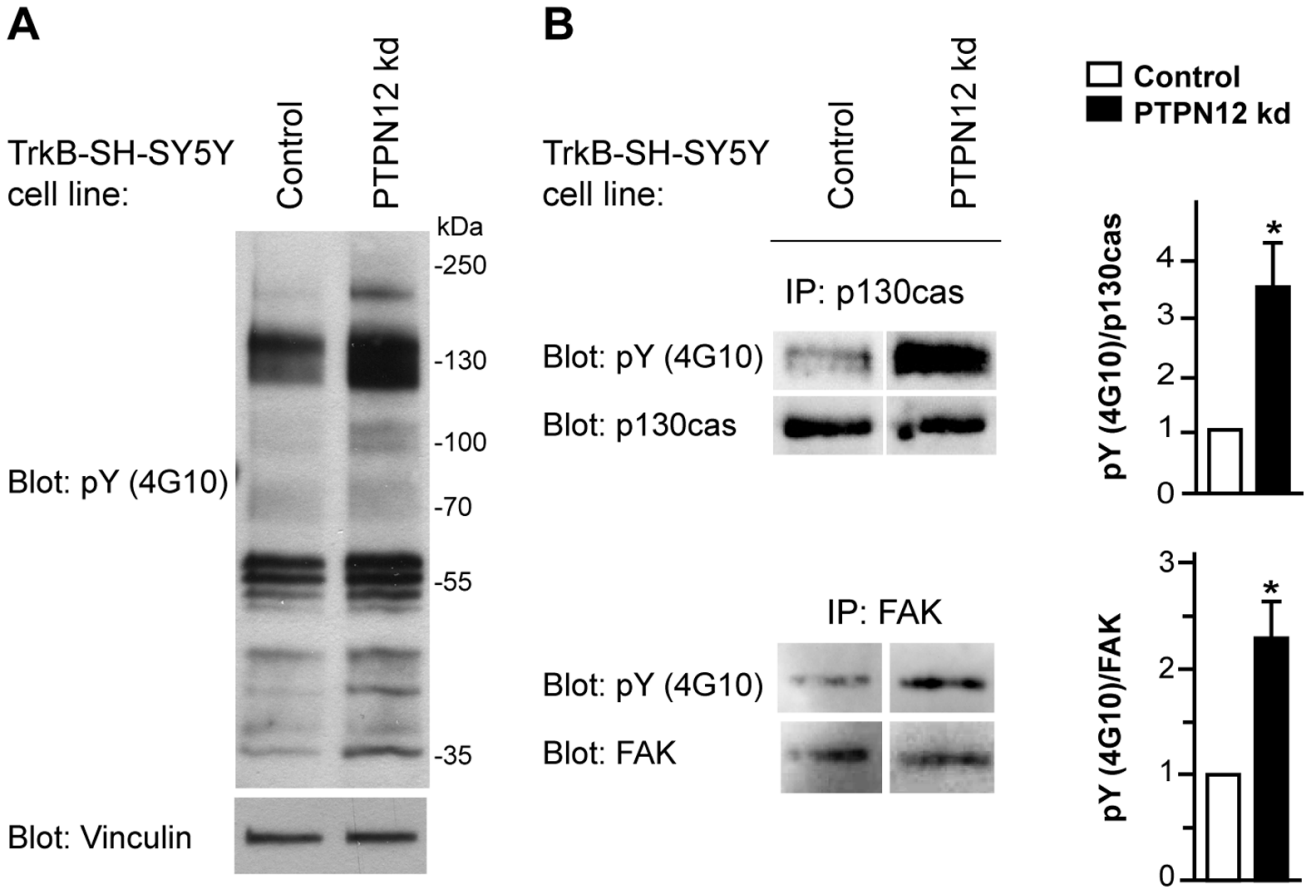
PTPN12 changes the phosphotyrosine profile in TrkB-SH-SY5Y cells. A) Total phosphotyrosine pY levels (4G10 antibody) were revealed by western blotting in lysates from control or PTPN12 kd cells cultured for 24 h and starved for 4 h prior to lysis (basal condition). Vinculin served as loading reference. B) Lysates prepared from cells as in A) were immunoprecipitated (IP) using p130cas or FAK antibodies and blotted for total pY levels (4G10 antibody). Total p130cas and FAK levels were used as the respective references. Densitometric quantification of the tyrosine phosphorylation level of p130cas and FAK (4G10), compared to total p130cas and FAK levels, is shown as mean and S.E.M. of three and five independent experiments respectively, with values normalized to the control. Statistical analysis was performed using Student’s paired t-test (*: p≤0.05).

PTPN12, an intracellular PTP, acts on many substrates related to motility in response to growth factors or ECM, such as Shc, p130cas, FAK, Csk, paxillin and PSTPIP1 [70]–[72]. We first compared the pattern of tyrosine phosphorylation between PTPN12 knockdown and control cells in the absence of BDNF. As shown in Figure 6A PTPN12 knockdown was associated with increased phosphotyrosine (pY) levels. Most prominent were bands with apparent MW ∼130 kDa, corresponding in size to the well-established PTPN12 substrates p130cas and FAK (MW∼125 kDa). Indeed, through immunoprecipitation experiments we showed that both p130cas and FAK are hyperphosphorylated on tyrosine residues in PTPN12 knockdown cells (Figure 6B) and thus most likely are substrates for PTPN12 in TrkB-SH-SY5Y cells.

### PTPN12 Modulates TrkB Signaling

**Figure 7:**
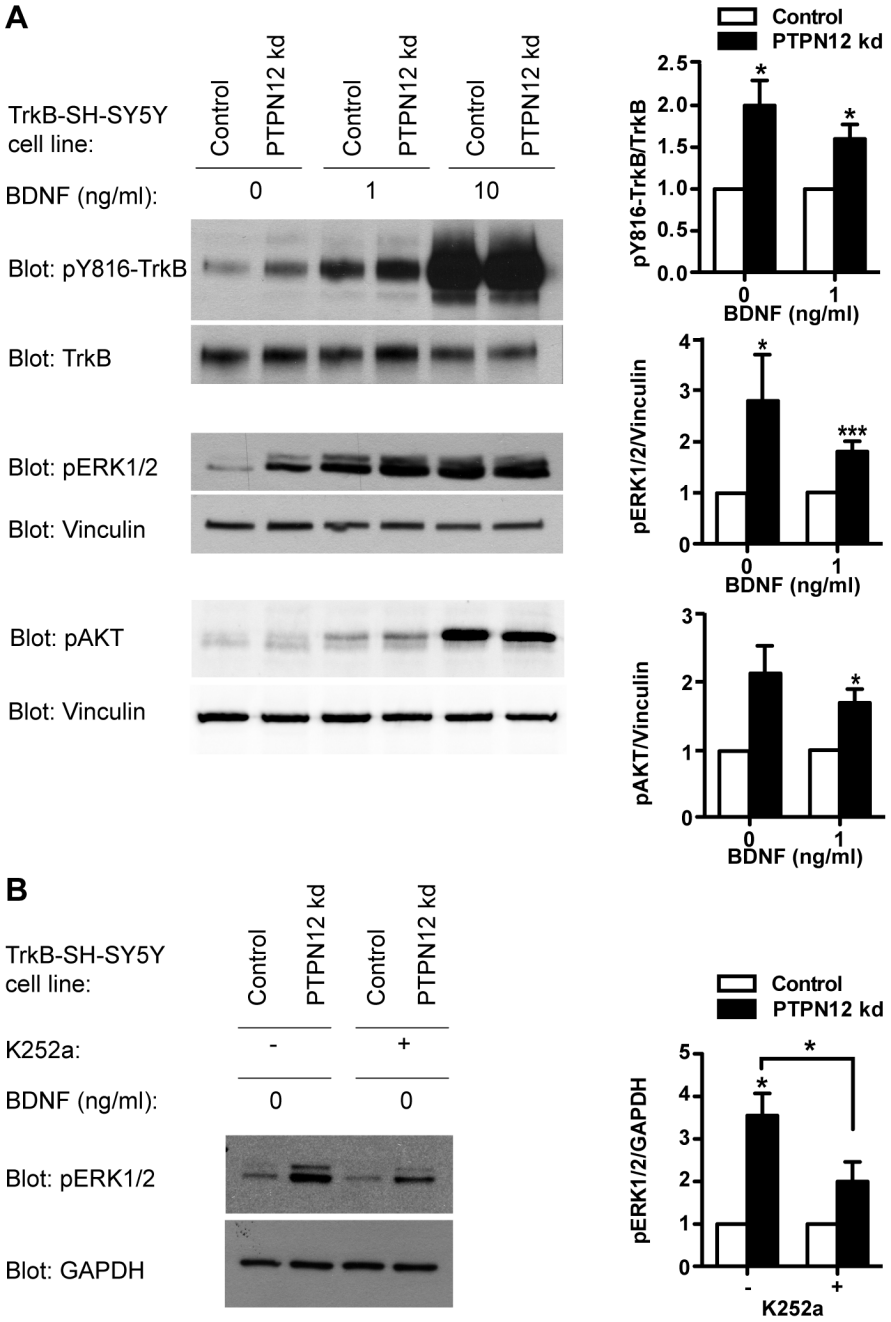
PTPN12 modulates TrkB signaling in TrkB-SH-SY5Y cells. A) Western blot analyses for pY816-TrkB, TrkB, pERK1/2, pAKT and Vinculin levels in control and PTPN12 kd cells cultured for 24 h, starved for 4 h and stimulated for 5 min with 0, 1 or 10 ng/ml BDNF. Graphs show densitometric quantifications of pY816-TrkB compared to total TrkB, and pERK1/2 and pAKT compared to total Vinculin, for cells in absence of BDNF (basal level) or with 1 ng/ml BDNF (mean and S.E.M. of three, seven, and four independent experiments respectively). PTPN12 kd values were normalized to the level of the control for each BDNF concentration. NB: p-value = 0.076 for comparison of pAKT between control and PTPN12 kd both at 0 ng/ml BDNF. B) PTPN12 kd and control cells cultured as in A) in the absence of BDNF ± K252a were analyzed for pERK1/2 levels using GAPDH as a reference. Respective densitometric quantifications are shown as mean and S.E.M. of three independent experiments; PTPN12 kd is normalized to the level of the control in the absence or presence of K252a. Statistical analyses were performed using Student’s paired t-test comparing control and Ptpn12 kd, except where otherwise specified (*: p≤0.05; ***: p≤0.005). NB: p-value = 0.060 for comparison of pERK between control and PTPN12 kd both treated with K252.

We wished to test the hypothesis that phosphorylation of TrkB itself would be affected by knockdown of PTPN12 in TrkB-SH-SY5Y cells. Recent data, in tumor contexts, have shown that PTPN12 can dephosphorylate receptor tyrosine kinases [64]. Indeed, a phospho-specific antibody against pY816 in TrkB [73] revealed increased tyrosine phosphorylation of this residue following PTPN12 knockdown, in absence of exogenous BDNF, or in presence of low BDNF concentrations, (Figure 7A). Quantification of pY816 in TrkB relative to total TrkB levels showed a ∼2-fold increase in pY TrkB in cells lacking PTPN12 in the absence of BDNF, and ∼1.5-fold increase in the presence of 1 ng/ml BDNF (Figure 7A).

The increase in TrkB activation following PTPN12 knockdown was accompanied by increased phosphorylation of the MAPK ERK1/2 (Figure 7A), in accordance with ERK1/2, a regulator of differentiation and neurite outgrowth, being a downstream mediator of TrkB signaling [1], [5], [13]; PTPN12 knockdown also boosted Akt phosphorylation (Fig. 7A).

ERK1/2 can be activated through changes in FAK and p130cas activity [74]. To elucidate the events upstream of ERK1/2 phosphorylation we treated cells under basal conditions with the Trk inhibitor K252a and quantified the ensuing effect on pERK1/2 levels (Figures 7B). K252a treatment reduced the increase in pERK1/2 phosphorylation caused by PTPN12 knockdown. This suggests (subject to the *caveat* of K252a specificity) that the pERK phenotype partly can be explained by changes related to TrkB, but also partly by TrkB-independent events, such as increased p130cas and FAK activities.

**Figure 8:**
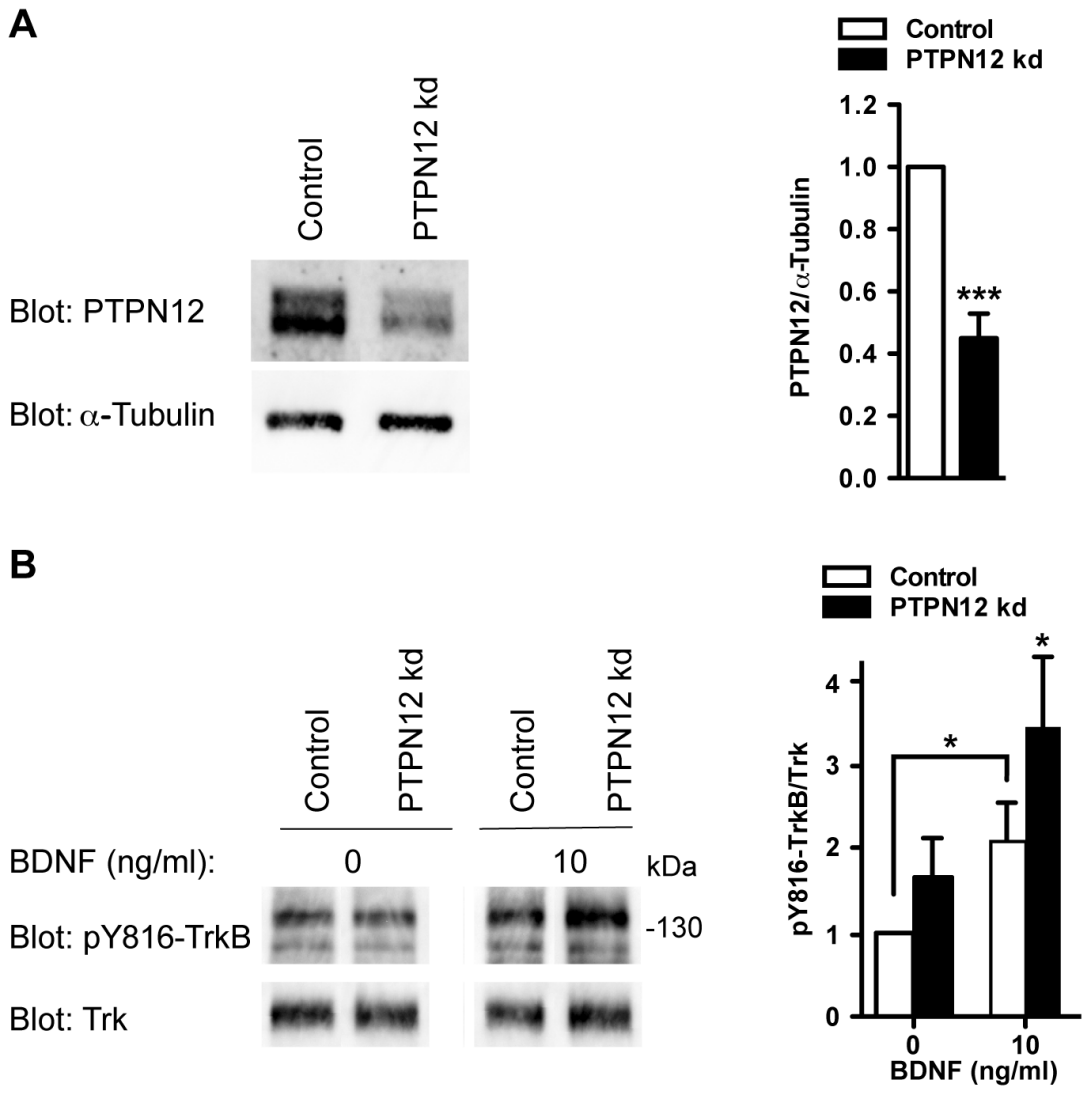
PTPTN12 knockdown in primary mouse hippocampal neurons enhances tyrosine phosphorylation of TrkB. A) Western blot analysis of PTPN12 expression in mouse hippocampal neurons expressing a pLKO-shRNA targeting PTPN12 (PTPN12 kd) or a non-silencing control. α-Tubulin is used as reference. Densitometric quantification of PTPN12 expression is shown as mean and S.E.M. of five independent experiments, and values are normalized to the level of the non-silencing control. B) Western blot analysis of pY816-TrkB and Trk levels in mouse hippocampal neurons expressing a pLKO-shRNA targeting PTPN12 (PTPN12 kd) or a non-silencing control. The neurons were cultured for 5 days and then stimulated for 5 min with 0 or 10 ng/ml BDNF. Densitometric quantification of pY816-TrkB compared to total Trk levels are shown as mean and S.E.M. of five independent experiments, and values are normalized to the level of the control in the absence of BDNF. Statistical analyses were performed using Student’s paired t-test comparing control and Ptpn12 kd, except when otherwise specified (*: p≤0.05; ***: p≤0.005).

To ask whether PTPN12 also controlled TrkB activation state in primary neurons, we subjected cultured mouse hippocampal neurons to lentiviral knockdown of endogenous PTPN12. Analogously to our observations in TrkB-SH-SY5Y cells, stable knockdown of Ptpn12 (Figure 8A) resulted in a statistically significant increase in TrkB phosphorylation on residue Y816 in response to exposure to BDNF (Figure 8B), confirming that Ptpn12 negatively regulates TrkB phosphorylation in cultured mouse primary neurons.

## Discussion

### Development of an Automated siRNA Screening Strategy in Neuronally Differentiated SH-SY5Y Cells

We developed a robust high-throughput siRNA-based screening protocol in differentiated human neuron-like SH-SY5Y cells to identify regulators of BDNF-TrkB signaling pathway controlling neurite outgrowth. Sequential treatment of SH-SY5Y cells with RA and BDNF creates a homogenous population of mature neuron-like cells that are withdrawn from the cell cycle, express many markers of mature neurons (such as GAP43, MAP2, NeuN, synaptophysin and TrkB), have neuronal marker enzyme activity (tyrosine- and dopamine-β-hydroxylases), exhibit carbachol-evoked noradrenaline release, and are dependent on NTs for survival [41], [42]. Accordingly, they are a reasonable alternative to primary neurons when it comes to large-scale RNAi-based screening methods, since they are more easily transfected to induce RNAi-mediated gene silencing than primary neurons [75].

Signaling events are dependent on the combined and regulated effects of both kinases and phosphatases. A recent study using over-expression of 450 selected kinases and phosphatases in hippocampal neurons revealed that only a few of the hits identified as enhancers of neurite outgrowth on laminin also enhanced outgrowth on poly-lysine [76], suggesting that the signaling pathways underlying neurite outgrowth are particular to the experimental conditions used. This is further underscored by the observation that, among the three reported large-scale siRNA screens investigating the function of kinases and/or phosphatases in relation to neurite outgrowth/migration, not a single protein consistently promoted or suppressed neurite outgrowth/migration [37], [38], [77] (compared in [76]). It is therefore crucial to perform screens that are carefully designed to answer particular biological questions. Our present study differed from all of the above in focusing on identification of phosphatases that regulate BDNF-mediated neurite outgrowth in fully differentiated neuronal cells. To our knowledge this is the first large-scale screen addressing signaling events directly related to NT-mediated neurite outgrowth.

### Neurite Outgrowth is Modulated by a Large Number of Diverse Phosphatases

We did not find any specific phosphatase gene families that were particularly enriched among the top positive or negative regulators identified by the primary screen. This is in accordance with previous observations that a diverse range of proteins are involved in regulation of neurite outgrowth, both across functional gene classes [37] and among kinase or phosphatase families [76], [77]. Most of the validated negative regulatory phosphatases (Figure 4), such as the lipid phosphatase ACYP1, the aspartate-based (HAD-family) phosphatase ENOPH1, and the tyrosine phosphatases PTPMT1 and PTPN12 are poorly characterized, in terms of association with neurite outgrowth. It will be of interest to investigate their relation to the classical BDNF-TrkB signaling pathway. Other negative regulators, such as TENC1, have better-known activities that could point to mechanisms of regulation of BDNF-TrkB signaling. For TENC1 these include decreasing plasma membrane phosphatidylinositol 3,4,5-trisphosphate levels [78], inhibition of Akt signal transduction [79], [80], activation of the growth cone collapse promoting protein Rho [81] and direct interaction with a RTK [82]. The strongest identified and validated positive regulator of BDNF-TrkB mediated neurite outgrowth, PPM1J, belongs to the PP2C phosphatase subgroup [83], [84] and is also very poorly characterized. Thus, further studies of its regulatory function in NT signaling are warranted.

Single gene studies have previously implicated PTPs in the control of BDNF responses, e.g. LAR (PTPRF) as a positive regulator of BDNF neurotrophic activity in hippocampal neurons [35], and MKP-1 (DUSP1) in BDNF-induced axon branching in cortical neurons [36]. The phosphatases RPTPζ (PTPRZ1) and RPTPσ (PTPRS) can counteract NGF/TrkA-induced outgrowth in cell types other than SH-SY5Y [33], [34]. Among these PTPs, our screen only detects LAR (PTPRF) (as a negative regulator of outgrowth; Table S1). Differences in read-out and cell type, and/or insufficient sensitivity of our siRNA-based protocol may explain the lack of detection by our screen of the above PTPs previously implicated in control of neurotrophin-induced neurite outgrowth.

### PTPN12 as a Negative Regulator of Neurite Outgrowth

Among the so-called “canonical” protein tyrosine phosphatases we found PTPN12 to be the most effective negative regulator of BDNF-TrkB mediated outgrowth in our screen. We selected this gene for further mechanistic follow-up, given the almost complete absence of studies in a neuronal context on this otherwise well-studied PTP. PTPN12 consists of an N-terminal catalytic domain and a C-terminal domain containing a proline-rich region to which many interaction partners bind (such as p130cas, FAK, paxillin, Grb2 and Csk), a NxPH binding site for Shc, and a CTH domain that interacts with PSTPIP [72].

PTPN12 has been previously linked to membrane protrusion, focal adhesion turnover and cell migration [60]–[63], [68], [85] through its effect on GEFs and GAPs for the small Rho GTPases Rac1 and RhoA, respectively [63], [86]. Neurite extension requires reorganization of the growth cone cytoskeleton and is largely controlled by Rho family GTPases [1], [87]. Rac1 and RhoA have antagonistic effects on this process, in that Rac1 promotes neurite outgrowth and RhoA triggers growth cone collapse [87], [88]. Thus, PTPN12 might partly control BDNF-TrkB mediated neurite outgrowth through regulation of Rac1 and RhoA activity. Indeed, several GEFs for Rac1 are recruited to activated Trk receptors in neurons, resulting in activation of Rac1 and neurite outgrowth [89], [90].

We found that PTPN12 knockdown led to an increase in tyrosine phosphorylation of both p130cas and FAK in TrkB-SH-SY5Y cells. This is in accordance with reports of hyperphosphorylated p130cas and FAK in *Ptpn12^−/−^* fibroblasts [60], and hyperphosphorylated p130cas in *Ptpn12^−/−^* embryos [85]. The role of p130cas in migration depends largely on its tyrosine phosphorylation by FAK and Src family kinases (SFKs) after growth factor receptor or integrin activation (by BDNF and collagen, respectively, in our assays) and assembly of the p130cas-Crk-DOCK180 scaffold, which drives localized Rac1 and MAPK activation [70], [74], [91]. Hyperphosphorylation of p130cas following PTPN12 knockdown could thus be caused indirectly through increased FAK activity, which could also contribute to the increase in pERK1/2 (Figure 7) known to be important for neurite outgrowth together with Rac1 activation [1].

Importantly, we have also shown that the TrkB receptor for BDNF becomes hyperphosphorylated on Y816 in absence of PTPN12 in TrkB-SH-SY5Y cells as well as in primary hippocampal neurons, pointing to a role for PTPN12 in controlling TrkB receptor proximal signaling events. This effect of PTPN12 on TrkB activation predominantly appears at non-saturating levels of exogenous BDNF. One possibility is that PTPN12 directly dephosphorylates TrkB, similarly to the ability of RPTPζ (PTPRZ1) and RPTPσ (PTPRS) to dephosphorylate TrkA, and thereby attenuate NGF-induced ERK1/2 signaling [33], [34]. PTPN12 co-precipitates with several other RTKs [65] and was recently shown to dephosphorylate EGFR and HER2 in breast tumors, thereby inhibiting MAPK signaling [64]. PTPN12 knockdown in human mammary epithelial cells led to a two-fold increase in RTK phosphorylation [64], which is in the same range of our observations for TrkB after PTPN12 knockdown in TrkB-SH-SY5Y cells (Figure 7) and in hippocampal neurons (Figure 8). PTPN12 could be recruited to TrkB through its capacity to interact with adaptors or scaffold proteins such as Grb2, Crk, and Shc present at the activated TrkB receptor [1], [72]. Alternatively, PTPN12 might not directly dephosphorylate TrkB, but could affect its phosphorylation indirectly, e.g. through its effects on intermediate tyrosine kinases, such as members of the Src family (which are known to regulate NT-independent transactivation of Trk receptors [15], [58], [92], [93]), or through FAK. Experiments with PTPN12 substrate trapping mutants may help clarify these issues [94]. Finally, the issue of possible effects of PTPN12 on the control of phosphorylation of TrkA and TrkC will also need to be considered.

Interestingly, PTPN12 knockdown does not affect neurite outgrowth in absence of BDNF in SH-SY5Y cells (Figure S5), but does so in (TrkB-overexpressing) TrkB-SH-SY5Y cells (Figure 5B). This may indicate that PTPN12 might modulate TrkB phosphorylation only when TrkB is itself activated (by ligand in SH-SY5Y cells, or by overexpression in TrkB-SH-SY5Y cells); mechanisms responsible could include altered TrkB or PTPN12 localization, or modulation of PTPN12 catalytic activity in response to BDNF/TrkB activation.

The phosphorylated Y816 residue in TrkB acts as a PLCγ binding site, whereas phosphorylation of another residue, Y515, is associated with Shc docking. The literature indicates that it is not possible to cleanly assign ERK activation to one site: there appear to be Y515/Shc-independent pathways to ERK activation, and the Y816/PLC-γ-binding site can contribute to ERK activation by Trks [95], [96]. We have also observed statistically significant effects of PTPN12 knockdown on phosphorylation of the Y515 residue (FN, VD, and JS, unpublished observations), a docking site for Shc. Thus, a causal link between PTPN12 knockdown-induced TrkB hyperphosphorylation and ERK hyperactivation seemed plausible. To investigate the contribution of TrkB hyperphosphorylation to the pERK1/2 activation associated with PTPN12 knockdown, we used the Trk inhibitor K252a. K252a treatment reduced the PTPN12 knockdown-induced increase in pERK1/2 by only 50%, suggesting that ERK activation following PTPN12 knockdown is only partly due to TrkB hyperactivation. The presence of hyperphosphorylated p130cas and FAK, associated with increased integrin responsiveness, might account for the remaining part of the elevated ERK phosphorylation following PTPN12 knockdown (subject to the *caveats* associated with the use of inhibitors). At any rate it may be exceedingly difficult, and of limited utility, to try and further disentangle both processes, as neuronal FAK and Src are integration points between growth factor and integrin signaling, with activation of both pathways having synergistic effects on neurite outgrowth [97], [98].

### Outlook

BDNF participates in regenerative processes [25], [26], and decreased BDNF function is associated with neurodegeneration, cognitive decline, and psychiatric disorders [20]–[24]. Our study reveals the extent of phosphatome regulation of BDNF responsiveness. Phosphatases that negatively control BDNF sensitivity may have promise as pharmacological targets to potentiate the effect of limiting amounts of endogenous factor. While the diversity in surface structure properties among PTPs (including e.g. the predicted presence of an accessible secondary non-catalytic substrate-binding pocket in PTPN12 [99]) might augur well for development of selective PTP inhibitors, and progress has been made, substantial hurdles remain to be overcome [100], [101].

Strikingly, while we identify PTPN12 as a novel negative regulator of BDNF-receptor activity, we observe that such “classical” PTPs only play a limited role in phosphatase control of this tyrosine kinase-mediated response (8/60 = 13% of the hits under the p<0.01 significance mark). This prompts a comprehensive view of phosphatase control, with multiple categories of evolutionarily unrelated phosphatase families having converged to orchestrate a biological response [102], [103]. Our study thus reveals the breadth of the spectrum of target families that could potentially be exploited to modulate BDNF signaling to therapeutic effect.

## Materials and Methods

### Cell Culture, Differentiation, Stimulation and siRNA Transfection of SH-SY5Y Cells

The SH-SY5Y neuroblastoma cell line was from S. Påhlman (Lund University, Malmø, Sweden). Cells were grown on dishes pre-coated with 50 µg/ml of type I rat tail collagen in Minimum Essential Medium (MEM, Gibco) supplemented with 2 mM L-glutamine, 10% fetal bovine serum (FBS) (Gibco), penicillin (100 units/ml), and streptomycin (100 µg/ml) (pen-strep) (MEM medium). For experiments, cells were seeded at low density (13,000 cells/cm^2^) in medium additionally supplemented with 10 µM *all*-*trans* retinoic acid (RA) (Sigma) (RA medium). After 4–5 days, cells were washed twice in serum-free MEM medium and incubated with BDNF (a gift from Regeneron Pharmaceuticals Inc., Tarrytown, NY, USA) in serum-free MEM medium as indicated. siRNA transfections were carried out after 3 days in RA medium. Briefly, medium was changed into RA medium without antibiotics, and a transfection mixture consisting of OptiMEM (Invitrogen), siRNA (final concentration 50 nM) and Lipofectamine 2000 (Invitrogen), prepared according to the manufacturer’s instructions, was added. After 8 h, transfection medium was replaced with fresh RA medium. A SH-SY5Y cell line expressing full-length TrkB (TrkB-SH-SY5Y cells) was a kind gift from A. Schramm (Essen, Germany) [69]. TrkB-SH-SY5Y cells were grown on collagen-coated dishes in RPMI-1640 (Gibco) supplemented with 2 mM L-glutamine, 10% FBS, pen-strep (RPMI medium) and geneticin (500 µg/ml). For experiments, cells were plated for 24 h in RPMI medium, starved for 4 hours in serum-free RPMI, and stimulated with BDNF in serum-free RPMI medium as indicated. Trk inhibitor K252a (300 nM) (Sigma) was applied as a 15 min pre-treatment and left in medium throughout the experimental period. Cells were maintained at 37°C in a humidified incubator containing 5% CO_2_.

### Generation of Lentiviral Particles and Infection of TrkB-SH-SY5Y Cells and Primary Hippocampal Neurons

A 2^nd^ generation packaging system was used to produce virus. 5 µg transfer vector was co-transfected together with 10 µg pMD2G (VSV-G, Addgene plasmid 12259) and 5 µg psPax2 (gag-pol, tat-rev; Addgene plasmid 12260), using calcium phosphate coprecipitation, into 2.6×10^6^ human embryonic kidney 293T cells (ATCC CRL-11268) seeded 24 h before transfection. Supernatant containing viral particles was harvested 48 h post-transfection.

To infect TrkB-SH-SY5Y cells we used GIPZ-based lentiviral shRNAmir transfer vectors targeting human *PTPN12* (sense: CGAGTTAAATTGACATTAA) or the non-silencing control (Open Biosystems). Unconcentrated supernatant containing viral particles was used directly for infection. The genetically modified cells were grown in puromycin (1 µg/ml) to maintain the presence of the transfer vector.

For lentiviral knockdown in mouse hippocampal neurons, we used a variant of the pLKO.1-puro lentiviral vector (Sigma), in which the puro^R^ marker was replaced by an eGFP-WPRE-encoding fragment (introduced between the Acc65 and BamHI sites). The sense targeting sequence for mouse *Ptpn12* was GCCCTAAAGGTTGATGATGTA and for the non-targeting control CAACAAGATGAAGAGCACCAA (Sigma TRC1.5 clone SHC002). Viral supernatant was concentrated (100-fold) by ultracentrifugation at 80,000×*g* for 2 h at 4°C in conical tubes using the SW32 Ti rotor (Beckman). The viral pellet was resuspended in PBS, aliquoted and stored at −80°C. Titer was estimated by infecting 293T cells with virus dilutions and quantification of GFP positive cells by flow cytometry (BD FACSCalibur) after 4 days.

#### Culture, lentiviral transduction, and analysis of primary hippocampal neurons

Animal work was conducted in accordance with relevant national and international guidelines, specifically as issued by the French Ministry of Agriculture, and approved by *Direction Départementale des Services Vétérinaires de Paris* (Paris departmental directorate of veterinary services). Animals were sacrificed by cervical dislocation. E16 fetal mouse brains were dissected, dissociated and cultured largely as described in [104]. Briefly, after trypsin and DNAse treatment, 1.5×10^5^ primary hippocampal neurons were seeded in 12-well plates pre-coated with poly-D-lysine (Sigma) in neurobasal medium supplemented with 1X B27 (Life Technologies), of which half was replaced every 2–3 days. Lentiviral transduction was performed 60–90 min after plating at a multiplicity of 5. After 5 days in culture, infected neurons were stimulated with BDNF as indicated for 5 min before lysis.

#### Cell lysates and immunoprecipitation

SH-SY5Y cells were seeded in 24-well plates (NUNC) in RA medium and transfected with siRNAs after 3 days. 48 h post-transfection, cells were changed into serum-free MEM medium with 10 ng/ml BDNF and left to differentiate as indicated. Cells were washed twice in PBS and lysed in a lysis buffer containing 62.5 mM Tris-HCl pH 6.8, 2% SDS, 10% glycerol, 1 mM DTT, 0.025% bromophenol blue, 1 mM Na_3_VO_4_, 50 mM NaF, 10 µg/ml leupeptin, 10 µg/ml aprotinin and 1 mM PMSF. Lysates were boiled for 5 min and equal volumes used directly for western blotting. TrkB-SH-SY5Y cells were grown and stimulated as described, washed twice with cold PBS, and lysed on ice in RIPA buffer (50 mM Hepes pH 7.4, 150 mM NaCl, 1% Triton-X-100, 1% sodium deoxycholate, 0.1% SDS, 1 mM EGTA, 1 mM Na_3_VO_4_, 50 mM NaF, 10 µg/ml leupeptin, 10 µg/ml aprotinin, and 1 mM phenylmethylsulfonyl fluoride (PMSF)). Lysates were cleared by centrifugation at 12,000 rpm for 20 min at 4°C and protein concentration quantified with the bicinchoninic acid protein assay (Pierce). Immunoprecipitation of Trk receptors was carried out with magnetic dynabeads (Invitrogen) using 500 µg protein and an anti-Trk antibody (sc-11, Santa Cruz Biotechnology). C57BL/6 mice were bred and kept at conventional animal facilities at the University of Copenhagen. Lysates from whole brain homogenates were prepared using TM buffer (Chemicon). Cerebellar granule neurons prepared as previously described [105] were cultured for 3 days *in vitro* and lysed in RIPA buffer. Hippocampal neurons were lysed in 50 mM Hepes pH 7.5, 150 mM NaCl, 1.5 mM MgCl_2_, 1 mM EDTA, 10% Glycerol, 1% Triton X-100, 1 mM Na_3_VO_4_, 10 mM NaF, 4% Protease Inhibitor Cocktail (Roche), and 1 mM PMSF.

#### Western blotting

Equal amounts of protein were resolved by standard SDS-polyacrylamide gel electrophoresis and transferred to Immobilon PVDF membrane (Millipore). Membranes were blocked in PBS-Tween (0.1%) and 5% non-fat dry milk powder for 1 h at room temperature and incubated overnight at 4°C in primary antibodies: anti-p130cas (#610271), anti-FAK (#610088) and anti-TRKB (#610101) (all from BD Transduction Laboratories), anti-TRK (C-14) (#sc-11 from Santa Cruz Biotechnology, INC.), anti-GAP43 (#NB300-143 from Novus Biologicals), anti-GAPDH (#G8795), anti-Vinculin (#V9131) and anti-α-Tubulin (#T9026) (all from Sigma), anti-PTPN12 (a gift from Dr. M. Tremblay, Department of Medicine, Division of Experimental Medicine, McGill University, Montreal, Canada), anti-phosphoTrkB (Y816) (a gift from Dr. M. Chao, Skirball Institute, New York University School of Medicine), anti-p-44/42 MAPK (ERK1/2) (#9101) and anti-p-AKT (Ser 473) (#4051) (both from Cell Signaling), anti-ROCK I (#sc-6055 from Santa Cruz Biotechnology) or anti-phosphot-yrosine 4G10 (prepared as a hybridoma culture supernatant). Blots were subsequently incubated for 1 h at room temperature with horseradish peroxidase-conjugated secondary antibodies (Jackson Immunoresearch Laboratories), and developed with an enhanced chemiluminiscence detection system (PerkinElmer Life Sciences or Thermoscientific). Band intensities were quantified with ImageJ or Fusion software. To assay more than one protein on the same membrane, membranes were incubated in stripping buffer (62.5 mM Tris pH 6.78, 2% SDS, 100 mM mercaptoethanol) for 45 min at 60°C before reblotting.

#### High-throughput neurite outgrowth screen

SH-SY5Y cells were seeded into twelve 96-well plates (NUNC) in RA medium and transfected 3 days later with the siRNA library. 48 h post-transfection, cells were changed into serum-free MEM medium with 10 ng/ml of BDNF, and left to differentiate for 3 more days. Cells were then fixed in 3.5% paraformaldehyde and 0.5% glutaraldehyde in PBS for 20 min at 4°C, permeabilized with 0.2% Triton-X-100, and stained with an antibody to β-III-tubulin (Sigma T6880; 1∶300) overnight at 4°C in 3% BSA in PBS. The next day an Alexa594-conjugated goat-anti-mouse antibody (Molecular Probes; 1∶400) containing Hoechst 33342 (Molecular Probes, 0.5 µg/ml) was applied for 1 h at room temperature. After washing cells were left in PBS. Plating of cells and medium changes were performed manually, while transfection, fixation and staining of cells were performed automatically using a Hamilton Microlab STAR Liquid Handling Station. The screen was performed three times using different passages of the SH-SY5Y cell line.

#### siRNA library

A library targeting 254 phosphatases and various controls (including siRNAs targeting TrkB (*NTRK2*), as well as negative controls) was custom-designed, based on comprehensive literature and database mining. Three siRNAs/gene were designed by and ordered from Ambion. The gene list and siRNA sequences used are available upon request. The library was delivered in 3×384-well plates and dissolved to a concentration of 1.2 µM, and was at the time of transfection of siRNAs automatically distributed out into 12 96-well plates. As controls for positive and negative modulation of neurite outgrowth, functionally validated siRNAs targeting TrkB or ROCK1 were spotted manually into the 384-well plates (resulting in their presence twice in each 96-well plate). For validation experiments, siRNAs including controls were purchased from Sigma.

#### Automated image analysis for neurite outgrowth

15 images/well were acquired using an IN Cell Analyzer 1000 automated high-throughput microscope (GE Healthcare) installed with a Nikon 20× objective, and the images were automatically analyzed using an algorithm designed using the IN Cell Developer software (GE Healthcare). Briefly, nuclei were first segmented and counted using the Hoechst channel. Then a segmentation mask was superimposed over cell bodies and their associated neurites using the β–III-tubulin channel. A representative cell body size was then approximated using random images from control wells and subsequently subtracted from the ‘cell body/neurite’ bitmap to generate a ‘neurite’ bitmap. Neurite length was determined using the ‘fiber length’ parameter of the IN Cell Developer software, which measures the total length within a single fibrous shape. Finally, neurite length/cell was calculated by dividing the total measured neurite length by the number of nuclei in each well. Typically at least 800 cells/well were counted. Neurite-like morphology of TrkB-SH-SY5Y cells was analyzed automatically with the IN Cell Analyzer 1000 workstation 3.5 software package using the form factor profile, where 1 is a completely round cell and cells with a form factor >3.5 had various long protrusions from the cell morphologically resembling neurites. Cells with a form factor >3.5 were scored as having neurite-like protrusions.

#### Data normalization

Activity (neurite length per cell) was first normalised to the plate median and further to the median row activity of the particular screen to control for edge effects observed across the rows. Following normalisation the average activity for each sequence-specific siRNA over the three screens was calculated and used for subsequent statistical analysis. Due to the three-fold redundancy of the siRNA library, three activity values per gene were used for the statistical analysis.

#### Statistical analysis

Statistical analysis was carried out using redundant siRNA activity (RSA) analysis as described in [55]. Two different analyses were carried out: one for identification of positive regulators and one for identification of negative regulators (see Materials and Methods Document S1 for details).

## Supporting Information

Figure S1 **Effect of RA/BDNF differentiation of SH-SY5Y cells on GAP43 protein levels.** Non-differentiated (ND) SH-SY5Y cells, cells treated with RA for 5 or 8 days, and cells treated for 5 days with RA followed by 3 days with BDNF (50 ng/ml) were evaluated for GAP43 protein levels by Western blotting using GAPDH as a reference. Densitometric quantification of GAP43 compared to GAPDH levels is shown as mean and S.E.M. of three independent experiments with GAP43 and GAPDH values normalized to the level of the control without RA and BDNF (5d ND). Statistical analysis was performed using Student’s paired t-test (*: p≤0.05).
(TIF)

Figure S2 **Transfection efficiency in RA/BDNF differentiated SH-SY5Y cells.** A) Cells were plated, treated and transfected with siGLO siRNA according to the assay outline in Figure 2A. Transfection efficiency was evaluated by flow cytometry analysis 24 h post-transfection. Red and blue lines represent non-transfected and transfected cells, respectively. B) Lipofectamine was left out of the transfection reaction to verify that the high transfection efficiency observed was not caused by siGLO siRNA sticking to the surface of the transfected cells (same line coloring as in A).
(TIF)

Figure S3 **Test of scrambled siRNAs at different BDNF concentrations.** The neurite outgrowth assay was performed as described in Figure 2A with six different scrambled (SCR) siRNAs. Control refers to non-transfected cells, while mock refers to cells transfected without siRNA. Transfections were tested at 0, 10 and 50 ng/ml BDNF. Data are shown as mean and S.E.M. of four replicates.
(TIF)

Figure S4 **siRNA-mediated knockdown efficiency in RA/BDNF differentiated SH-SH5Y cells.** Four hits randomly selected among the ones chosen for validation were evaluated for siRNA-mediated knockdown efficiency. Cells were plated, treated and transfected as described in Figure 2A. 72 h post-transfection (and 24 h after addition of 10 ng/ml BDNF), mRNA expression was analyzed by qPCR. Data are normalized to *HRPT1* expression levels and shown relative to the UNC1 scrambled control, as mean and S.E.M. of triplicates. The experiment is representative of two independent experiments.
(TIF)

Figure S5 **Validation of hits at different BDNF concentrations.** Hits selected for validation were tested for their effect in the absence of BDNF (A and B), or in the presence of a saturating BDNF concentration (50 ng/ml) (C and D), as opposed to 10 ng/ml BDNF used in the screen and the primary validation. UNC1 is the scrambled siRNA control while ROCK1 or TrkB targeting siRNAs served as biological controls. A and C: 11 negative regulators; B and D: 4 positive regulators. Red and green indicate negative and positive regulators, respectively, and filled and open bars represent validated and non-validated hits, respectively. Data are shown as mean and S.E.M. of triplicates (*: p≤0.05; **: p≤0.01; and ***: p≤0.001; using one-way ANOVA followed by Dunnett’s multiple comparison test with UNC1 as reference).
(TIF)

Figure S6 **Neurite outgrowth of TrkB-SH-SY5Y cells in response to BDNF.** A) TrkB expression in different cells and tissue. Lysates from SH-SY5Y cells differentiated for 5 days with RA, non-treated TrkB-SH-SY5Y cells, mouse whole brain homogenate and mouse cerebellar granule neurons (CGNs) cultured for 3 days *in vitro* were evaluated for TrkB expression using western blotting. Vinculin was used as reference. B) TrkB-SH-SY5Y cells were plated for 24 h, followed by 3 days in the absence or presence of 50 ng/ml BDNF. Phase contrast pictures were taken at 20× magnification. Scale bar = 20 µm. C) Cells treated as in B) were evaluated for GAP43 expression using western blotting with GAPDH as reference.
(TIF)

Table S1 **RSA analysis of negative regulators of BDNF-TrkB mediated neurite outgrowth.**
(XLSX)

Table S2 **RSA analysis of positive regulators of BDNF-TrkB mediated neurite outgrowth.**
(XLSX)

Document S1 **Supporting Materials and Methods.**
(DOCX)
