# Supplementary figures and images for: A Loss-of-Function Screen for Phosphatases that Regulate Neurite Outgrowth Identifies PTPN12 as a Negative Regulator of TrkB Tyrosine Phosphorylation
Source: PLoS One. 2013 Jun 13;8(6):e65371. doi: 10.1371/journal.pone.0065371 (PMC3681791; doi:10.1371/journal.pone.0065371)

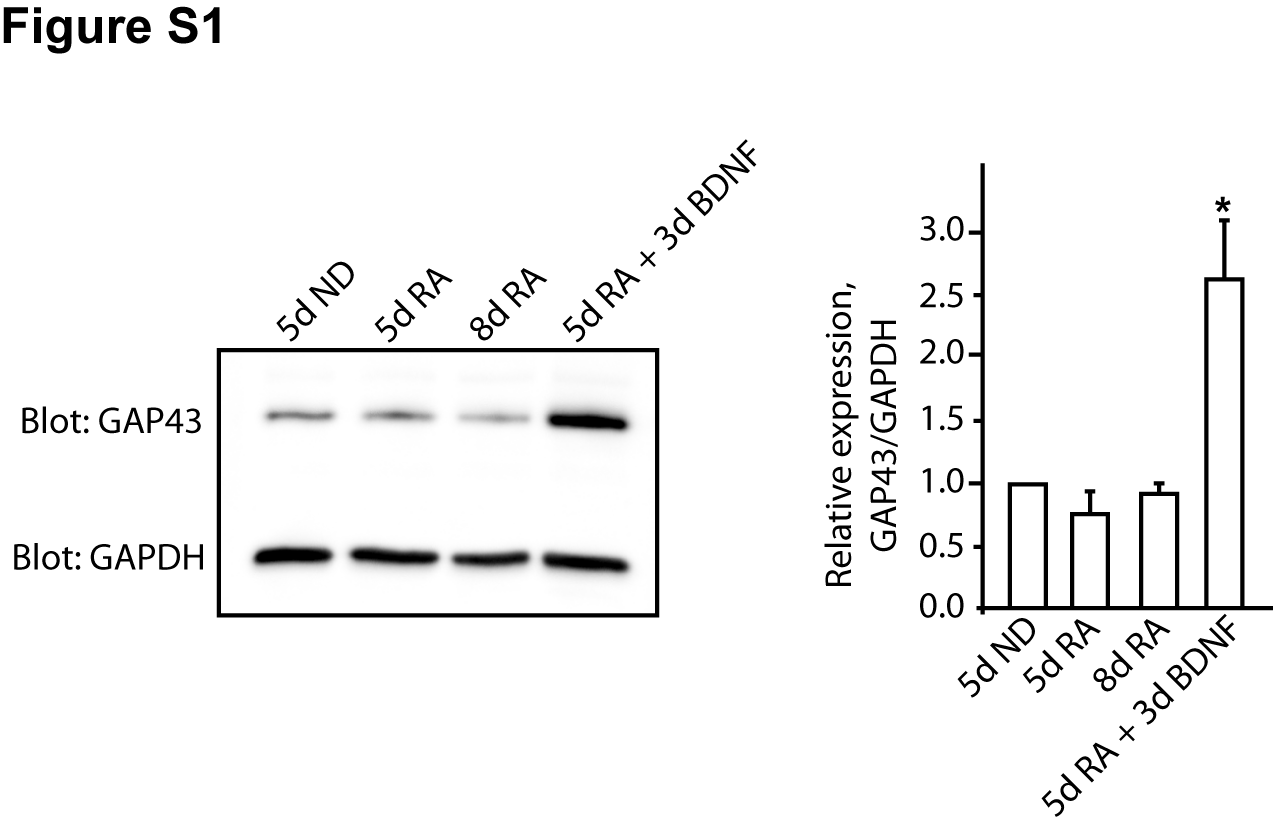

Supplement: Figure S1 — Effect of RA/BDNF differentiation of SH-SY5Y cells on GAP43 protein levels. Non-differentiated (ND) SH-SY5Y cells, cells treated with RA for 5 or 8 days, and cells treated for 5 days with RA followed by 3 days with BDNF (50 ng/ml) were evaluated for GAP43 protein levels by Western blotting using GAPDH as a reference. Densitometric quantification of GAP43 compared to GAPDH levels is shown as mean and S.E.M. of three independent experiments with GAP43 and GAPDH values normalized to the level of the control without RA and BDNF (5d ND). Statistical analysis was performed using Student’s paired t-test (*: p≤0.05). (TIF) [file pone.0065371.s001.tif]

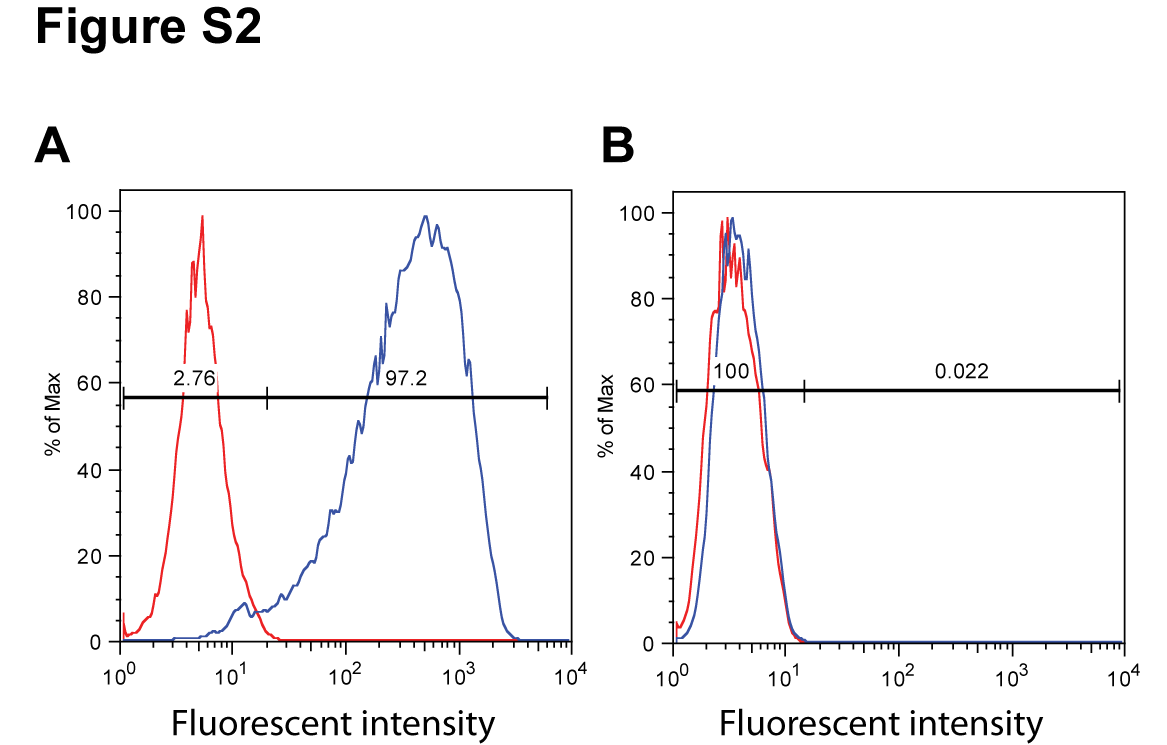

Supplement: Figure S2 — Transfection efficiency in RA/BDNF differentiated SH-SY5Y cells. A) Cells were plated, treated and transfected with siGLO siRNA according to the assay outline in Figure 2A. Transfection efficiency was evaluated by flow cytometry analysis 24 h post-transfection. Red and blue lines represent non-transfected and transfected cells, respectively. B) Lipofectamine was left out of the transfection reaction to verify that the high transfection efficiency observed was not caused by siGLO siRNA sticking to the surface of the transfected cells (same line coloring as in A). (TIF) [file pone.0065371.s002.tif]

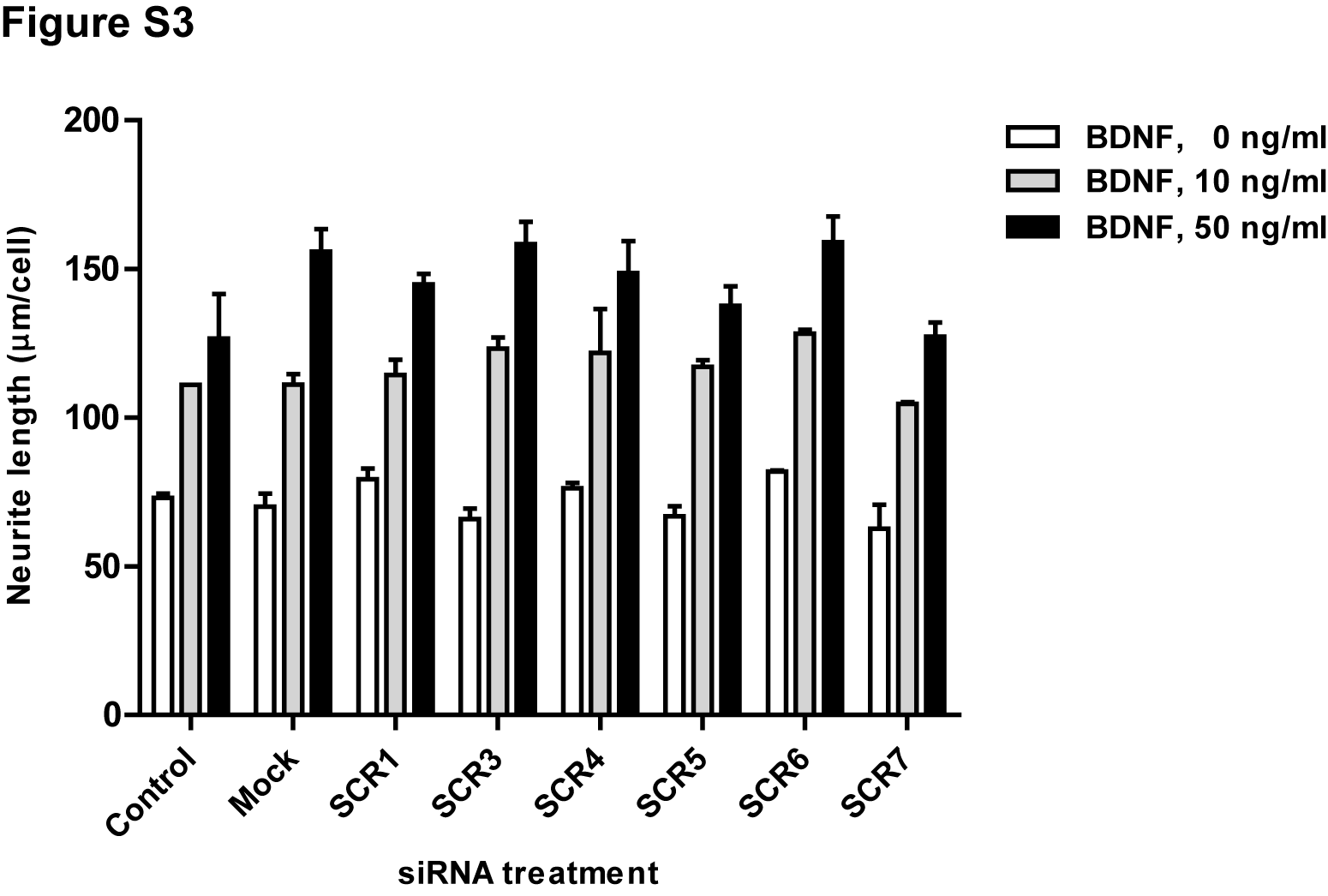

Supplement: Figure S3 — Test of scrambled siRNAs at different BDNF concentrations. The neurite outgrowth assay was performed as described in Figure 2A with six different scrambled (SCR) siRNAs. Control refers to non-transfected cells, while mock refers to cells transfected without siRNA. Transfections were tested at 0, 10 and 50 ng/ml BDNF. Data are shown as mean and S.E.M. of four replicates. (TIF) [file pone.0065371.s003.tif]

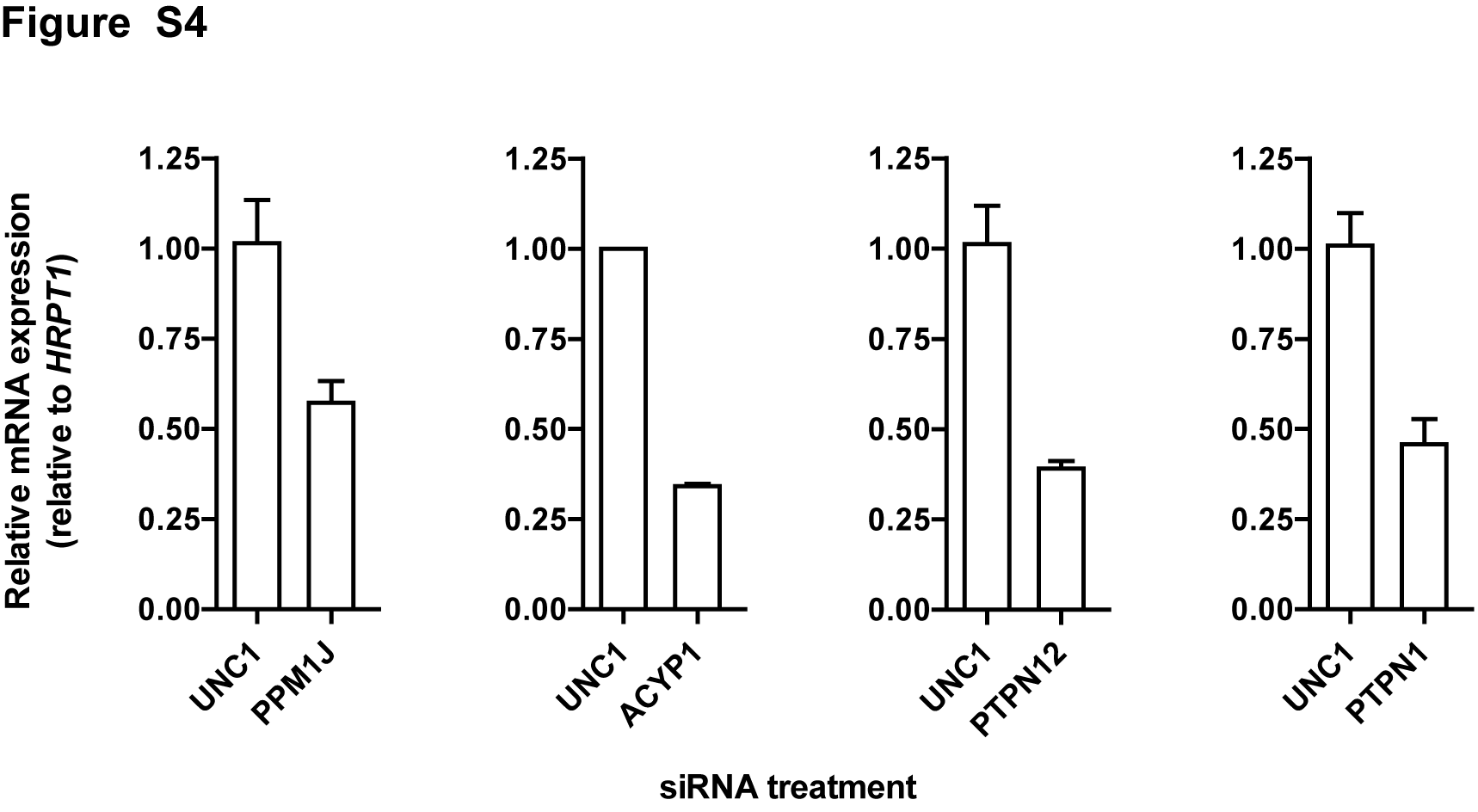

Supplement: Figure S4 — siRNA-mediated knockdown efficiency in RA/BDNF differentiated SH-SH5Y cells. Four hits randomly selected among the ones chosen for validation were evaluated for siRNA-mediated knockdown efficiency. Cells were plated, treated and transfected as described in Figure 2A. 72 h post-transfection (and 24 h after addition of 10 ng/ml BDNF), mRNA expression was analyzed by qPCR. Data are normalized to HRPT1 expression levels and shown relative to the UNC1 scrambled control, as mean and S.E.M. of triplicates. The experiment is representative of two independent experiments. (TIF) [file pone.0065371.s004.tif]

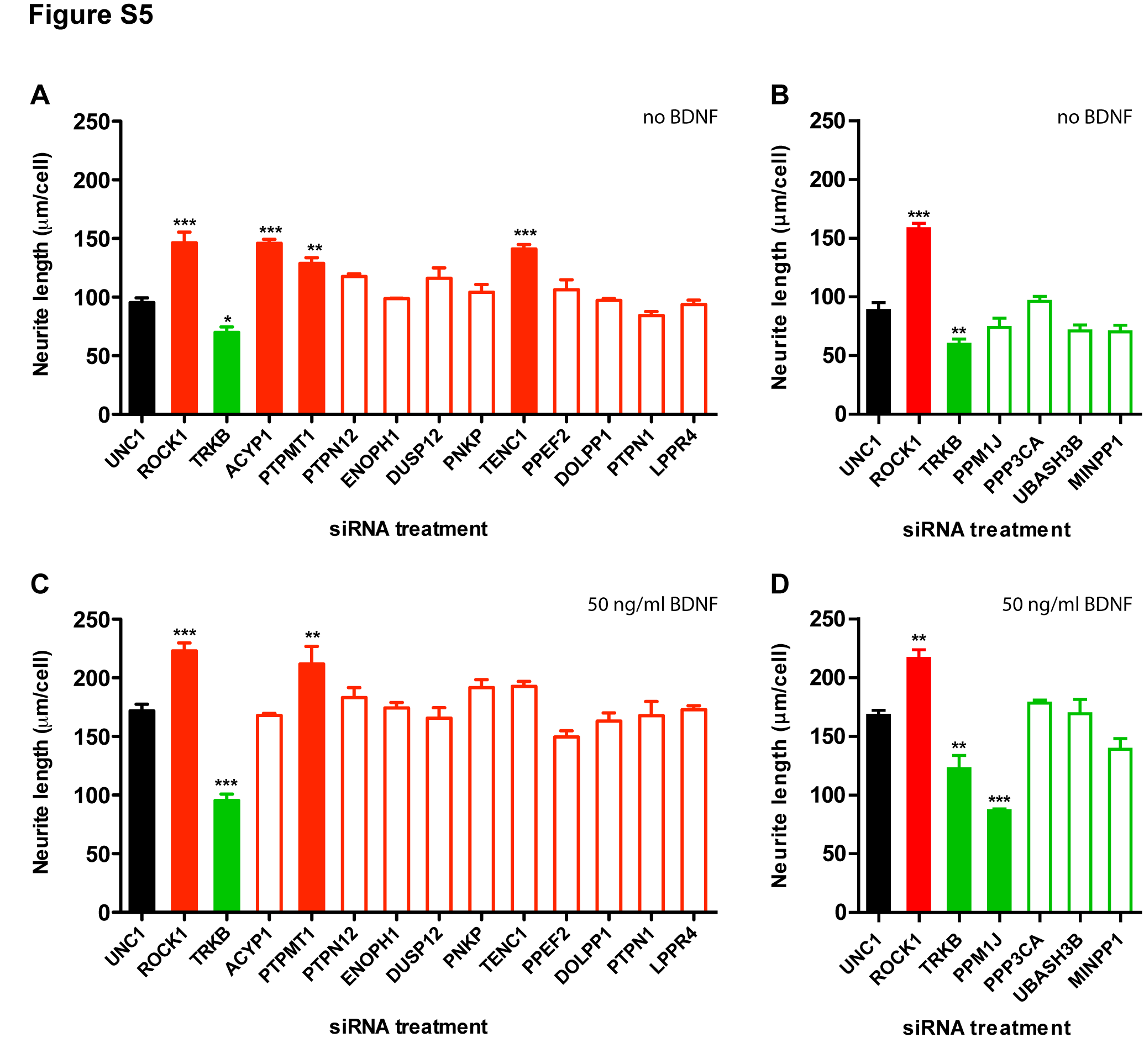

Supplement: Figure S5 — Validation of hits at different BDNF concentrations. Hits selected for validation were tested for their effect in the absence of BDNF (A and B), or in the presence of a saturating BDNF concentration (50 ng/ml) (C and D), as opposed to 10 ng/ml BDNF used in the screen and the primary validation. UNC1 is the scrambled siRNA control while ROCK1 or TrkB targeting siRNAs served as biological controls. A and C: 11 negative regulators; B and D: 4 positive regulators. Red and green indicate negative and positive regulators, respectively, and filled and open bars represent validated and non-validated hits, respectively. Data are shown as mean and S.E.M. of triplicates (*: p≤0.05; **: p≤0.01; and ***: p≤0.001; using one-way ANOVA followed by Dunnett’s multiple comparison test with UNC1 as reference). (TIF) [file pone.0065371.s005.tif]

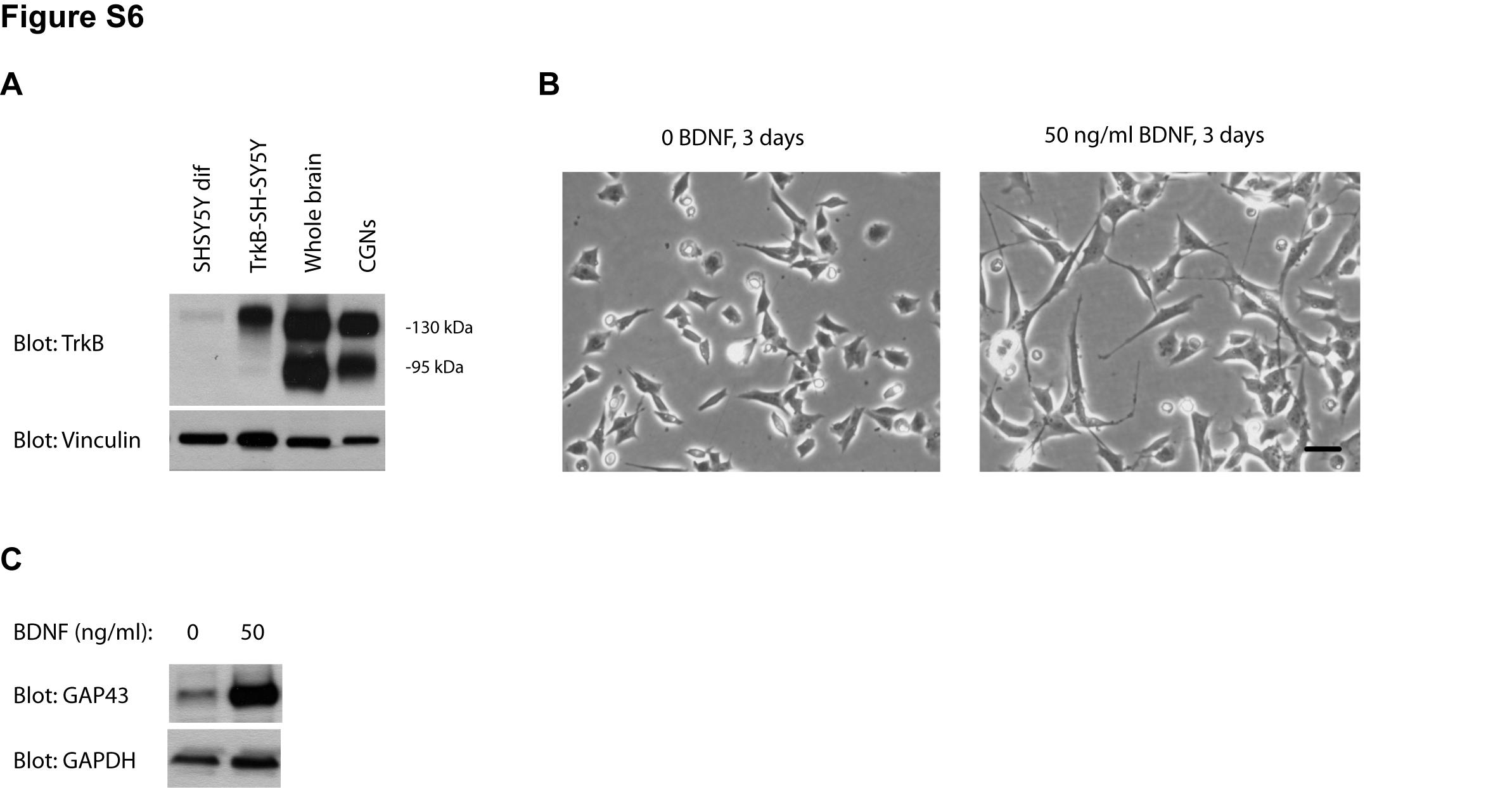

Supplement: Figure S6 — Neurite outgrowth of TrkB-SH-SY5Y cells in response to BDNF. A) TrkB expression in different cells and tissue. Lysates from SH-SY5Y cells differentiated for 5 days with RA, non-treated TrkB-SH-SY5Y cells, mouse whole brain homogenate and mouse cerebellar granule neurons (CGNs) cultured for 3 days in vitro were evaluated for TrkB expression using western blotting. Vinculin was used as reference. B) TrkB-SH-SY5Y cells were plated for 24 h, followed by 3 days in the absence or presence of 50 ng/ml BDNF. Phase contrast pictures were taken at 20× magnification. Scale bar = 20 µm. C) Cells treated as in B) were evaluated for GAP43 expression using western blotting with GAPDH as reference. (TIF) [file pone.0065371.s006.tif]
