# Supplementary material for: A Loss-of-Function Screen for Phosphatases that Regulate Neurite Outgrowth Identifies PTPN12 as a Negative Regulator of TrkB Tyrosine Phosphorylation
Source: PLoS One. 2013 Jun 13;8(6):e65371. doi: 10.1371/journal.pone.0065371 (PMC3681791; doi:10.1371/journal.pone.0065371)
Supplement: Document S1 — Supporting Materials and Methods. (DOCX) [file pone.0065371.s009.docx]

**SUPPORTING MATERIALS AND METHODS**

*qPCR.* Total RNA was isolated using RNeasy (Qiagen). The RT-PCR reaction was performed using the High Capacity cDNA reverse transcription kit (Applied Biosystems), and quantitative PCR using a 7300 Real Time PCR System (Applied Biosystems) was performed with 10 ng cDNA and 100 nM of the following primers: *PPM1J*: for 5'- TGGGATGTCACTACTGACTG and rev 5'- GCTGTGAGAGGAGTAAGTATGG, *PTPN12 (PTP-PEST)*: for 5'- CTGAAAGAACTCCTGAATCGT and rev 5'- ATATCTGTGGCTTCTGTTGG, *PTPN1*: for 5'- AACAGAGTGATGGAGAAAGG and rev 5'- GAGTTTCTTGGGTTGTAAGGT, *ACYP1*: for 5'- GAAGACAAGGAGGGTTTGAG and rev 5'- CAAGCCATTCCTGCATATGAC, *HRPT1*: for 5'- GCC AGA CTT TGT TGG ATT TG and rev 5'-CTC TCA TCT TAG GCT TTG TAT TTT G. The quantitative PCR data were analysed according to the 2^-ΔΔCt^ method [[1](#_ENREF_1)].

*Flow cytometry****.*** SH-SY5Y cells cultured in 6-well plates (NUNC) and differentiated with RA were transfected with siGLO siRNA (Dharmacon). As a control, cells were also transfected with siGLO siRNA in the absence of Lipofectamine 2000. 24h post-transfection cells were recovered by trypsinization and fixed with 1% PFA. Transfection efficiency was then analysed by flow cytometry on a FACSCalibur (BD Biosciences).

*Statistical analysis (detailed information).* The statistical analysis was carried out using redundant siRNA activity (RSA) analysis as described in [[2](#_ENREF_2)]. The analysis is based on a principle that takes into account the collective activity of multiple siRNAs targeting a specific gene, thus including the effect of potentially moderately functioning siRNAs. The method is in contrast to the widely used “high-activity cut-off based” approach for identification of hits in that it identifies siRNAs/hits distributed much deeper into the dataset (i.e. with lower activity values). RSA analysis has proven to have a much better validation efficiency/reconfirmation rate than the “high-activity cut-off based” approach. This is due to the contribution of more siRNA sequences to the p-value assigned to a specific gene (for most genes), thus reducing the chances of selecting false positive activities, resulting from either off-target effects or experimental and logistic artefacts, for validation [[2](#_ENREF_2)].

RSA analysis is based on the cumulative hypergeometric probability distribution. The analysis identifies hits by assigning a p-value to each gene. This p-value is the probability of observing a test statistics as extreme as, or more extreme than, the one actually observed, if the null hypothesis is indeed true. In the RSA analysis carried out the null hypothesis can be defined as the observed activity rank distribution being caused by chance. A low p-value for a gene therefore indicates that the distribution of one or multiple gene-specific siRNAs toward the top ranking slots is unlikely to occur by chance and therefore most likely can be attributed to a true biological activity of that gene.

First, all sequence-specific siRNAs are assigned a rank based on their activity in the screen (neurite length per cell). Two separate analyses are carried out for identification of either positive or negative regulators, and thus two different ranking lists are made with either lowest or highest activity ranked as 1. For each gene there are three gene-specific siRNAs: s1, s2 and s3. For each of these the probability for its observed rank position, r1, r2 and r3, is calculated. In terms of the cumulative hypergeometric distribution this is the probability that if you randomly pick r1, r2 or r3 siRNAs from the total number of siRNAs, respectively, you get at least the number of gene-specific siRNAs corresponding to its local rank (which will always be 1, 2 and 3). We thus end up with three probabilities, p1, p2 and p3 - one for each gene-specific siRNA, s1, s2 and s3. The lowest value of p1, p2 and p3 is assigned to *all* the 3 siRNAs and is the gene-specific p-value. The local rank of the siRNA from which the p-value was determined is named the critical rank. From the critical rank we can determine outliers as siRNAs (s1, s2, or s3), which have a rank worse than the siRNA with the critical rank. In addition, all siRNAs with activities below the median screen activity, set to 1, are automatically listed as outliers.

As an illustration we will look at ACYP1, which is the top gene among the negative regulators of neurite outgrowth. The corresponding three siRNAs are ranked as 8, 71 and 107 based on their activity in the screens. Their local ranks are simply 1,2 and 3.

a) First we look at the siRNA ranked 8. We calculate the probability that by randomly picking 8 siRNAs out of the total pool of 981 siRNAs we get at least one of the three siRNAs targeting ACYP1. We denote this probability by p1.

b) Secondly, for the siRNA ranked 74 we calculate the probability that by randomly picking 74 siRNAs out of the total pool of 981 siRNAs we get at least two of the three siRNA targeting ACYP1. We denote this probability by p2.

c) Last, for the siRNA ranked 107 we calculate the probability that by randomly picking 107 siRNAs out of the total pool of 981 siRNAs we get all of the three siRNAs targeting ACYP1. We denote this probability by p3.

The result is p1=0.0242, p2=0.049 and p3=0.0013. Since p3 is the lowest p-value, it is assigned to all the three ACYP1 gene-specific siRNAs and hence the gene. The critical rank is therefore 3 (see Figure 3B). If p3 would have been 0.1 instead of 0.0013 due to a lower activity rank, p1 would now be the lowest p-value, and both s2 and s3 would be outliers with only s1 contributing to the p-value assigned to ACYP1. The critical rank would have been 1 in this case.

RSA analysis does not necessarily favour genes, which are targeted by more siRNAs than other genes. Thus, a gene that is targeted by 3 relatively potent siRNAs will score higher by RSA analysis than a gene, which is targeted by 1 potent and 6 inactive siRNAs. In general, if many potent siRNAs with different sequences are contributing to a given gene-specific p-value, the biological evidence for that gene having a true function is more compelling than if the p-value is based on only one or a few siRNAs. In our dataset all genes tested are represented with three-fold redundancy, while the validated controls are represented 24 times. Even though the controls are conferring an artificial biological input to the RSA analysis (they are functionally validated identical siRNA sequences spotted multiple times), they still behave in the analysis as any other siRNA, but due to the high number of well-functioning control siRNAs compared to the three-fold redundancy of the phosphatase library, the p-values obtained for the controls are correspondingly much smaller. However, if the controls were not performing as expected (irrelevant of their large number), they would not rank among the top activity siRNAs for positive (NTRK2/TrkB) and negative (ROCK1) regulators and therefore not show up as significant regulators of neurite outgrowth. From our screen data it is clear that siRNA numbers *per se* does not determine the p-value, as TrkB *only* shows as a positive regulator, while ROCK1 *only* shows as negative regulator.

1. Livak KJ, Schmittgen TD (2001) Analysis of relative gene expression data using real-time quantitative PCR and the 2(-Delta Delta C(T)) Method. Methods 25: 402-408.

2. Konig R, Chiang CY, Tu BP, Yan SF, DeJesus PD, et al. (2007) A probability-based approach for the analysis of large-scale RNAi screens. Nat Methods 4: 847-849.
